# Naoxintong Capsule Alternates Gut Microbiota and Prevents Hyperlipidemia in High-Fat-Diet Fed Rats

**DOI:** 10.3389/fphar.2022.843409

**Published:** 2022-03-21

**Authors:** Yihang Lu, Haofang Wan, Yujia Wu, Jiehong Yang, Li Yu, Yu He, Haitong Wan, Chang Li

**Affiliations:** Key Laboratory of TCM Encephalopathy of Zhejiang Province, Zhejiang Chinese Medical University, Hangzhou, China

**Keywords:** Naoxintong capsule, high fat diet, gut microbiota, hyperlipidemia, short chain fatty acid

## Abstract

**Background:** Naoxintong Capsule (NXT) is a formulated Traditional Chinese Medicine (TCM) widely applied in the treatment of cardiovascular and metabolic diseases, most of which are closely related to hyperlipidemia as a major risk factor. Given the current limited understandings to the role of gut microbiota in the lipid-lowering effect of NXT and other TCM products, this study investigated the regulation of gut microbiota and lipid metabolism by NXT, and their potential relationship.

**Methods:** The chemical components of NXT were firstly analyzed with HPLC-MS method. In high fat diet (HFD)-fed rat models, as well as normal rats as control, the histopathological and biochemical changes of serum and liver were examined, including total cholesterol (TC), triglyceride (TG), low density lipoprotein cholesterol (LDL-C) and high density lipoprotein cholesterol (HDL-C). In addition, the gut microbiota community was analyzed using 16S rRNA sequencing technique, the fecal levels of gut microbiota related metabolites, including bile acids (BAs) and short chain fatty acids (SCFAs) were determined with HPLC-MS. The correlations of the clinical indicators and gut microbiota related indicators were then investigated statistically.

**Results:** The results showed that NXT exerted potential preventive effect on hyperlipidemia. Specifically, NXT significantly reduced the body weight, TC, TG and LDL-C in serum, increased HDL-C in serum, reduced the TC and TG in liver, as well as protected liver. The body weight, serum lipid levels and liver function were all significantly alleviated. The gut microbiota of the HFD-fed rats was reconstituted with supplementation of NXT. The fecal levels of gut microbiota related metabolites, including BAs and SCFAs were also altered. The correlation between the gut microbiota and clinical/metabolomic parameters was then studied. As the result, the amount of propionic aicd, *Firmicutes*/*Bacteroidetes* ratio (F/B) and the relative abundance of *Collinsella* in feces are the most possibly potential therapeutic biomarkers of NXT.

**Conclusion:** NXT was effective in regulation of gut microbiota and prevention of hyperlipidemia in HFD fed rats. The present work might provide novel insights into the anti-hyperlipidemia effect of TCM and afford new scientific evidence for clinical application of TCM.

## Graphical Abstract

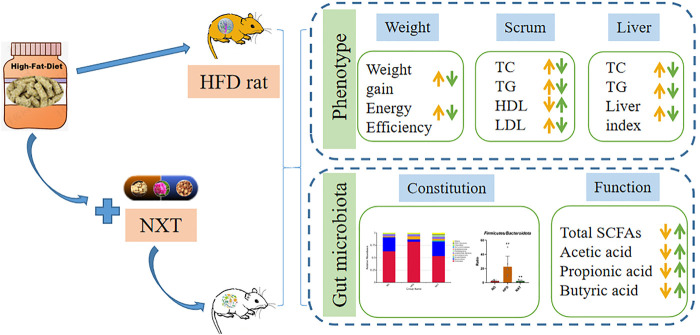

## Introduction

Hyperlipidemia is one of the major risk factors of atherosclerosis and related cardiovascular diseases, including stroke, myocardial infarction, sudden cardiac death and hypertension (Jia et al., 2021). Epidemiological studies revealed that hyperlipidemia is also closely associated with diabetes and non-alcoholic fatty liver disease (NAFLD) (Chalasani et al., 2018). According to 2019 statistics, the prevalence of dyslipidemia in China has exceeded 43%, most of which are hyperlipidemia (Opoku et al., 2019). In clinic, hyperlipidemia is usually characterized by abnormal lipid levels. Especially, increased serum levels of TC, TG and LDL-C, together with decreased level of HDL-C are the most significant indication for the diagnosis of hyperlipidemia. With the rapid rise in the number of patients with hyperlipidemia, effective therapeutic and preventive treatments of hyperlipidemia are in great demand (Wu et al., 2019). Besides the widely used lipid-lowering small molecular drugs such as fibrates and statins, phytomedicine, especially TCM, has shown great potential in the prevention and treatment of hyperlipidemia (Tong et al., 2018).

The gut microbiota plays a crucial role in human health (Chen et al., 2013). In recent years, emerging evidences have been accumulated to reveal the potential interaction between hyperlipidemia and gut microbiota (Jia et al., 2021). In particular, the significant rise of the ratio of *Firmicutes*/*Bacteroidetes* is considered as one of the golden proofs that the gut microbiota composition is greatly affected by high-fat diet and hyperlipidemia (Shin et al., 2014). The disorders of gut microbiota community can also lead to the disturbance of enterohepatic circulation, which will further exacerbate the dyslipidemia both in rats (Bäckhed et al., 2004) and human beings (Bäckhed et al., 2005). Therefore, modulation of the gut microbiota has become a beneficial strategy to regulate the lipid metabolism and further prevent the development of hyperlipidemia (Huang et al., 2020). For instance, Shi *et al.* reported that intake of α-amylase inhibitor enriched-extract from white common beans (*Phaseolus vulgaris* L.) could significantly reduce the body weight gain and improve the serum lipid levels (Shi et al., 2020). 16S rRNA gene sequencing and metabolomic investigations verified that the gut microbiota composition of the HFD-fed rats was modulated. Particularly, the relative abundances of *Firmicutes* and *Proteobacteria* decreased and that of *Bacteroidetes* and *Akkermansia* increased. Functional analysis assigned that the putative SCFA producing bacteria was significantly enriched.

On the other hand, Buchang Naoxintong capsule (NXT) is a formulated TCM for the treatment of cardiovascular diseases (Xu et al., 2016) and diabetes (Yan et al., 2020) with widespread application in China for over 20 years. NXT is a China Food and Drug Administration (CFDA) approved TCM product developed from a classic TCM decoction, Bu-Yang-Huan-Wu-Tang (Wang HY. et al., 2020). Typically, it consists of 16 different kinds of herbs, including *Astragalus mongholicus Bunge* (*Fabaceae*) (Huangqi), *Paeonia lactiflora Pall.* (*Paeoniaceae*) (Chishao), *Salvia miltiorrhiza Bunge* (*Lamiaceae*) (Danshen), *Angelica sinensis* (*Oliv*.) *Diels* (*Apiaceae*) (Danggui) and *Conioselinum anthriscoides* “*Chuanxiong*” (*Apiaceae*) (Chuanxiong). Previously reported chemical analysis with HPLC-MS method revealed that flavones, terpenoids and phenanthraquinones are the major components in NXT (Wang et al., 2015). Moreover, a lot of endeavor has been put into the pharmacological research on NXT. Diverse biological benefits of NXT were reported in previous studies, including antioxidant activity (Zhang et al., 2013), neuroprotectivity (Xue et al., 2016), lipid-lowering (Liang et al., 2018) and anti-inflammatory activities (Wang et al., 2017). It is noteworthy that several recent studies showed that NXT can also alleviate diseases by regulating the structure of gut microbiota, such as diabetes (Yan et al., 2020) and cardiovascular diseases (Zhang et al., 2019).

Herein, the anti-hyperlipidemia effect of NXT in HFD-fed rats was investigated. In particular, our study highlighted the regulation modes of NXT on the community structure and metabolites of gut microbiota.

## Materials and Methods

### Materials and Reagents

NXT capsules (batch No. 200313) were obtained from Shaanxi Buchang Pharmaceutical Co., Ltd. (Xianyang, China).

The normal-fat diet (ND, D12450H, 10% fat, 70% carbohydrate, 20% protein) and high-fat diet (HFD, D12451, 45% fat, 35% carbohydrate, 20% protein) were purchased from Jiangsu Xietong Pharmaceutical Bio-Engineering Co., Ltd. (Jiangsu, China) which were mixed and irradiated with cobalt-60 (radiation dose 25.0 kGy) before use.

Assay kits for TC, TG, HDL-C and LDL-C were purchased from Nanjing Jiancheng Bioengineering Institute (Nanjing, China). Assay kit for BCA was purchased from Shanghai Beyotime Biotechnology Co., Ltd. (Shanghai China). Assay kit for bile salt hydrolase (BSH; E.C.3.5.1.24) was purchased from Jiangsu Meibiao Biotechnology Co., Ltd. (Yancheng, China).

Methanol, acetonitrile and formic acid for HPLC analysis were all purchased from Tedia Co. Ltd. (Ohio, USA). Simvastatin (Simv, ≥98%) was purchased from Shanghai Rhawn Technology Development Co., Ltd. (Shanghai, China). Polyformaldehyde was purchased from Shanghai Macklin Biochemical Technology Co., Ltd. (Shanghai, China). Hematoxylin staining solution and eosin staining solution were purchased from Nanjing BioChannel Biotechnology Co., Ltd. (Nanjing, China). The water used was obtained with a Milli-Q water purification system (Bedford, USA).

### Major Component Analysis of the NXT With HPLC-MS

To qualitatively analyze the major components of NXT, the capsules were removed and 100 mg of the powder was precisely weighted. After 1 ml of 50% methanol was added, the powder was extracted under 200 W ultrasonic bath at 50°C. After 30 min extraction and subsequent centrifugation, the supernatant was collected for HPLC-MS analysis.

The HPLC analysis was carried out on a Thermo U3000 UHPLC system (Thermo, San Jose, United States) equipped with an auto-sampler, a dual pump, a column compartment and a diode array detector. A Waters SunFire C18 column (4.6 × 250 mm, 5 μm, Ireland) was employed. The mobile phase consisted of mobile phase A (0.5% aqueous formic acid) and mobile phase B (acetonitrile). The programmed gradient was as follows: 0–10 min, 2% B; 10–35 min, 2–10% B; 35–50 min, 10–15% B; 50–80 min, 15–25% B; 80–100 min, 25–60% B; 100–110 min, 60–90% B; 110–120 min, 90% B; 120–125 min, 90–2% B; 125–130 min, 2% B. The flow rate was 0.8 ml/min. The temperature of the column oven was maintained at 30°C. The injection volume was 10 μL. The chromatograms were recorded at 254 nm wavelength.

The high-resolution mass detection was performed on a Q-Exactive mass spectrometer (Thermo, San Jose, United States) equipped with an electrospray ionization (ESI) interface operated in negative ion mode. The ESI source parameters were optimized as follows: capillary voltage, 3.0 kV; sheath gas flow rate, 35 arb; auxiliary gas flow rate, 15 arb; sweep gas flow rate, 5 arb; capillary temperature, 300°C; probe heater temperature, 300°C; The mass spectra were acquired from m/z 100 to 1,000 in centroid mode, and the MS^2^ spectra were obtained with dd-MS^2^ mode with collision energy at 32 eV. Instrument control and data acquisition were achieved using Xcalibur software (Version 2.3.1, Thermo, San Jose, United States).

### Animals and the Experimental Design

All animal experiments were carried out strictly in accordance with the guidelines and regulations for the care and use of laboratory animals of Zhejiang Chinese Medicine University (10221). Male Sprague-Dawley (SD) rats with the body weights of (150 ± 20 g) were purchased from Shanghai Slac Laboratory Animal Co., Ltd. (Shanghai China). Rats were housed in a standardized pathogen free area with ambient temperature (25 ± 2°C), humidity (45 ± 5%) control, a 12 h light-dark cycle and standard laboratory animal feeding. After 7 days of acclimatization, the rats were randomly divided into five groups (*n* = 6) as follows: 1) ND group, fed with normal chow diet; 2) HFD group, fed with HFD; 3) NXT group, HFD-fed rats treated with normal dose of NXT (400 mg/kg/day, converted from maximum clinical dosage of NXT) by intragastric administration; 4) NXT-H group, HFD-fed rats treated with high dose of NXT (800 mg/kg/day, converted from twice the maximum clinical dosage) by intragastric administration; 5) Simv group, HFD-fed rats treated with simvastatin (10 mg/kg/day) by intragastric administration. The powder of NXT was dissolved in ultrapure water, and the suspension was given by gavage. Rats in ND and HFD groups received an equal volume of saline by gavage. Throughout the study, rats were fed freely while body weight and food intake were measured every week. In particular, energy efficiency was calculated as weight gain/energy intake (Wang Y. et al., 2020). After 6 weeks (Feng et al., 2019), all the rats were sacrificed after overnight fasting followed by collection of blood, liver and fecal samples. Samples of serum, feces and liver were stored at −80°C until the next analysis.

### Histopathological Analysis

Partial liver tissues were fixed in 4% formaldehyde solution for 24 h and then operated in standard for paraffin sections. The liver tissues were cut into 4-μm-thick sections and then stained with hematoxylin and eosin (H&E). Finally, the stained liver section was imaged with an optical microscope (Olympus, Tokyo, Japan) equipped with a digital camera. The liver index was calculated according to the following equation. Liver Index = Liver Weight (g)/Body Weight (g) × 100%.

### Biochemical Analysis

Serum levels of TC, TG, HDL-C, and LDL-C were quantified according to the manufacturer’s instructions using commercial enzymatic kits (Nanjing Jiancheng Bioengineering Institute, Nanjing, China). The liver tissue was homogenized in PBS firstly. Then the concentration of protein in liver homogenate was detected using BCA assay kits (Shanghai Beyotime Biotechnology Co., Ltd., Shanghai, China). The liver TC and TG were determined using the same kits. The results were expressed as mmol TC/TG per gram protein (mmol/gprot). All the measurements were performed on a microplate reader (Molecular Devices, San Francisco, United States).

### Measurement of the SCFAs in Feces

The SCFAs in the rat feces were quantitatively analyzed with a Thermo TRACE 1310-ISQ LT gas chromatography-mass spectrometer (Thermo, San Jose, United States). The rat feces (50 mg) were added to 100 μL (125 μg/ml) internal standard (isohexanoic acid), then diluted with 400 μL diethyl ether (including 50 μL 15% phosphoric acid), homogenized for 1 min and centrifuged at 4°C at 12,000 rpm for 10 min. Samples were injected with a split ratio of 10:1, and 1 μL of the filtered supernatant was injected into a gas chromatograph with flame ionization detector, Gas spectrometric conditions: One gas chromatographic column (Agilent HP-INNOWAX, 30 m × 0.25 mm, 0.25 μm, United States). The initial temperature was set to 90°C, then to 120°C at 10°C/min, then to 150°C at 5°C/min, and finally to 250°C at 25°C/min for 2 min. The injector and detector were set to 250 and 150°C respectively. The carrier gas was helium. The column flow rate was 1 ml/min, and the split ratio was 10:1.

### Measurement of the BAs and BSH in Feces

The rat feces (10 mg) were added to 1 ml methanol, homogenized for 2 min, and repeated twice. After ultrasonic bath for 30 min, the mixture was centrifuged at 12,000 rpm at 4°C for 10 min. Then 200 μL of the supernatant was added into 200 μL methanol, then the mixture was swirled for 30 s, and centrifuged at 4°C for 10 min at 12,000 rpm. The 300 μL supernatant was filtered with a 0.22 μm microfiltration membrane and used for analysis.

LC-MS analysis was performed on a Waters ACQUITY UPLC (Waters, Milford, United States) coupled with an AB SCIEX QTrap 4500 mass spectrometer (SCIEX, Framingham, United States). Chromatographic separation was carried out on ACQUITY UPLC BEH C18 column (2.1 × 100 mm, 1.7 μm, United States). The injection volume was 10 μL. The column temperature was 40°C. The mobile phase was consisting of mobile phase A (0.01% formic acid water) and mobile phase B (acetonitrile). The gradient elution conditions were 0–4 min, 25% B; 4–9 min, 25–30% B; 9–14 min, 30–36% B; 14–18 min, 36–38% B; 18–24 min, 38–50% B; 24–32 min, 50–75% B; 32–35 min, 75–100% B; 35–38 min, 100–25% B. The flow rate was 0.25 ml/min. Mass spectrometric conditions: one electrospray ionization (ESI) source and in negative ionization mode. The temperature of the ion source was 500°C. The voltage of the ion source was −4,500 V. The collision gas was 6 psi, the curtain gas was 30 psi, The atomizing gas and the auxiliary gas were 50 psi. The scan was performed using multiple response monitoring (MRM, Supplementary Table S2).

BCA kits (Shanghai Beyotime Biotechnology Co., Ltd., Shanghai, China) were used to determine the protein content in feces. BSH kits (Jiangsu Meibiao Biotechnology Co., Ltd., Yancheng, China) was used to measure the BSH activity in feces. Specific Activity of the BSH = BSH Activity/Protein Content (U/Gprot).

### Gut Microbiota Analysis

Total fecal microbial genome DNA was extracted with CTAB (Cetyl trimethylamine bromide). DNA concentration and purity were monitored on 1% agarose gel. The DNA was diluted to 1 ng/μL with sterile water.

PCR amplification of target fragment, forward primer 515F (5′-GTGCCAGCMGCCGCGGTAA-3′) and reverse primer 806R (5′-GGACTACHVGGGTWTCTAAT-3′) were used for PCR amplification of V3-V4 regions of bacterial 16S rRNA gene.

The mixture of PCR products was purified using the Qiagen Gel Extraction Kit (Qiagen, Germantown, United States). The TruSeq^®^ DNA PCR-Free Library Construction Kit (Illumina, San Diego, United States) was used to construct the sequencing library, and the index code was added. Finally, 250 bp sequencing of paired ends was performed using Illumina Novaseq platform.

FLASH (Version 1.2.7) was used to splice reads of each sample to get raw tags, and the QIIME (Version 1.7.0) quality control process was used to get clean tags. Using the UCHIME algorithm (UCHIME Algorithm) to connect the tag with the reference database (Silva database) for comparison to detect chimera sequences, which were removed to obtain effective tags. Sequence analysis was performed by Uparse software (Uparse v7.0.1001). Sequences with ≥97% similarity were assigned to the same operational taxonomy units (OTUs). For each representative sequence, the Silva Database was used to annotate the classification information based on the Mothur algorithm. In order to study the phylogenetic relationship between different OTUs and the differences of dominant species in different samples (groups), multiple sequence allocations were performed by Marken software (Version 3.8.31). OTUs abundance information was standardized using a standard sequence number corresponding to the sample with the least number of sequences. The alpha diversity was used to analyze the complexity of the species diversity in the sample, including observed species, chao 1, Shannon, Simpson, ACE, and good coverage, calculated by QIIME (Version 1.7.0) and displayed by R software (Version 2.15.3). The beta diversity of weighted and unweighted homogeneity was calculated by the QIIME software (Version 1.9.1). Principal component analysis (PCA) and Principal co-ordinates analysis (PCoA) analysis were shown in the R software (Version 2.15.3) using the WGCNA package, stat package, and ggplot2 package. Unweighted Pair-group Method with Arithmetic Means (UPGMA) Clustering was performed as a type of hierarchical clustering method to interpret the distance matrix using average linkage and was conducted by QIIME software (Version 1.9.1). Linear discriminant analysis (LDA) coupled with linear discriminant analysis effect size (LEfSe) was performed to identify the bacterial taxa differentially represented between groups at the genus or higher taxonomic levels, and was conducted by LEfSe software (LEfSe 1.0). The functional profiles of microbial communities were predicted by using phylogenetic reconstruction of unobserved states (PICRUSt). OTUs were picked using a closed reference (Greengenes ver. 13.5) at 97% sequence similarity, with normalization to control for differences in 16S rRNA copy number among OTUs. The relevant predicted genes and their function were aligned to KEGG database and the differences among groups were compared with the STAMP software (http://kiwi.cs.dal.ca/Software/STAMP). The two-side Welch’s *t*-test and Benjamini-Hochberg FDR correction were used in the between-group analysis. ANOVA with the Dunnett-Kramer test with the Benjamini-Hochberg correction were chosen for multiple-group analysis.

### Statistical Analysis

All data referenced above were expressed as the means ± SD and analyzed using GraphPad prism 8.0 (GraphPad, San Diego, CA, United States). The data was analyzed by one-way ANOVA, and the statistical differences between groups were verified by Dunnett multiple comparison test. *p* < 0.05 was considered to be significantly different.

## Results and Discussion

### Qualitive Analysis of Major Components in NXT With HPLC-HRMS

In accordance with previous reports (Wang et al., 2015), 14 major components of NXT were identified: uridine, guanosine, inosine, catechin, chlorogenic acid, paeoniflorin, hydroxysafflor yellow A, galloylpaeoniflorin, lithospermic acid, anhydrosafflor yellow B, rosmarinic acid, salvianolic acid A, salvianolic acid B and calycosin. The MS data of the components were summarized in Supplementary Table S1, together with the chromatogram of NXT at 254 nm in Supplementary Figure S1.

### NXT Reduces Body Weight Gain and Alleviates Related Parameters

**FIGURE 1:**
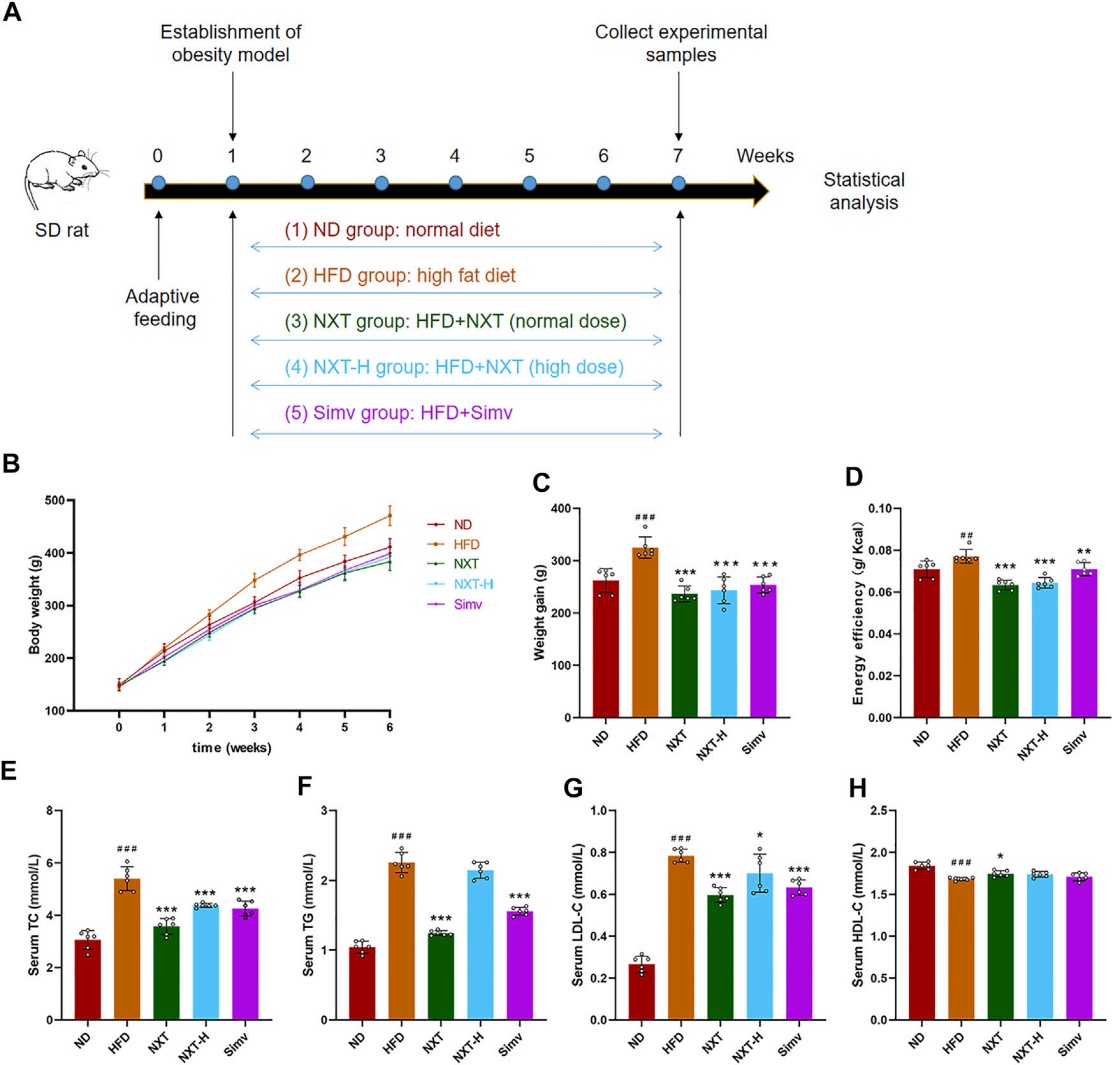
Effects of NXT on body weight gain and related parameters (*n* = 6 for each group). **(A)** Time-line diagram; **(B)** Body weight versus time analysis; **(C)** Body weight gain; **(D)** Energy efficiency; **(E–H)** Serum levels of TC, TG, LDL-C, and HDL-C. Data are presented as means ± SD, and analyzed using the one-way ANOVA test with Dunnett method. ^###^ *p* < 0.001, ^##^ *p* < 0.01 compared with ND group; ^*^ *p* < 0.05, ^**^ *p* < 0.01, ^***^ *p* < 0.001 compared with HFD group.

In order to evaluated the effect of NXT on the body weight gain and related parameters of HFD-treated rats, 30 male SD rats with similar body weight were randomly divided into five groups (*n* = 6) as shown in Figure 1A. It could be clearly observed that the average body weight of the HFD group was higher than that of the other groups all over the 6 weeks (Figure 1B). After the 6-weeks-treatment, the HFD group showed an extra 63.2 g weight gain compared with the ND group. It is also shown that supplementation of NXT or Simv can significantly reduce the body weight gain of HFD-fed rats. Noteworthily, the NXT group was slightly better than NXT-H and Simv groups (Figures 1B,C). The energy efficiency levels were in the same pattern as body weight gain (Figure 1D) where normal dose of NXT showed better effect comparing with the positive control Simv.

Next, we monitored the key biochemical parameters of serum to explore the effect of NXT against hyperlipidemia. As exhibited in Figures 1E–G, the serum levels of TC, TG, and LDL-C in HFD group were significantly higher (76.5%, 116.5%, and 195.0%) than those in ND group after 6 weeks. Supplementation with NXT apparently reduced the serum TC, TG, and LDL-C levels where normal dose of NXT exhibited better effects than high dose. In NXT group, the levels of the three markers were decreased by 28.6%, 45.8%, and 27.6%, respectively. It was noteworthy that NXT exhibited the same or even better effect on the serum lipid concentrations comparing with the popular lipid-lowering drug, Simv. Meanwhile, the serum HDL-C level in NXT group was significantly increased compared with HFD group, while the levels of NXT-H group and Simv group revealed no significant difference (Figure 1H). These results strongly proved the effect of NXT in alleviation of hyperlipidemia and hypercholesterolemia in HFD-fed rats.

### NXT Prevents Liver From HFD-Induced Adipose Hypertrophy

**FIGURE 2:**
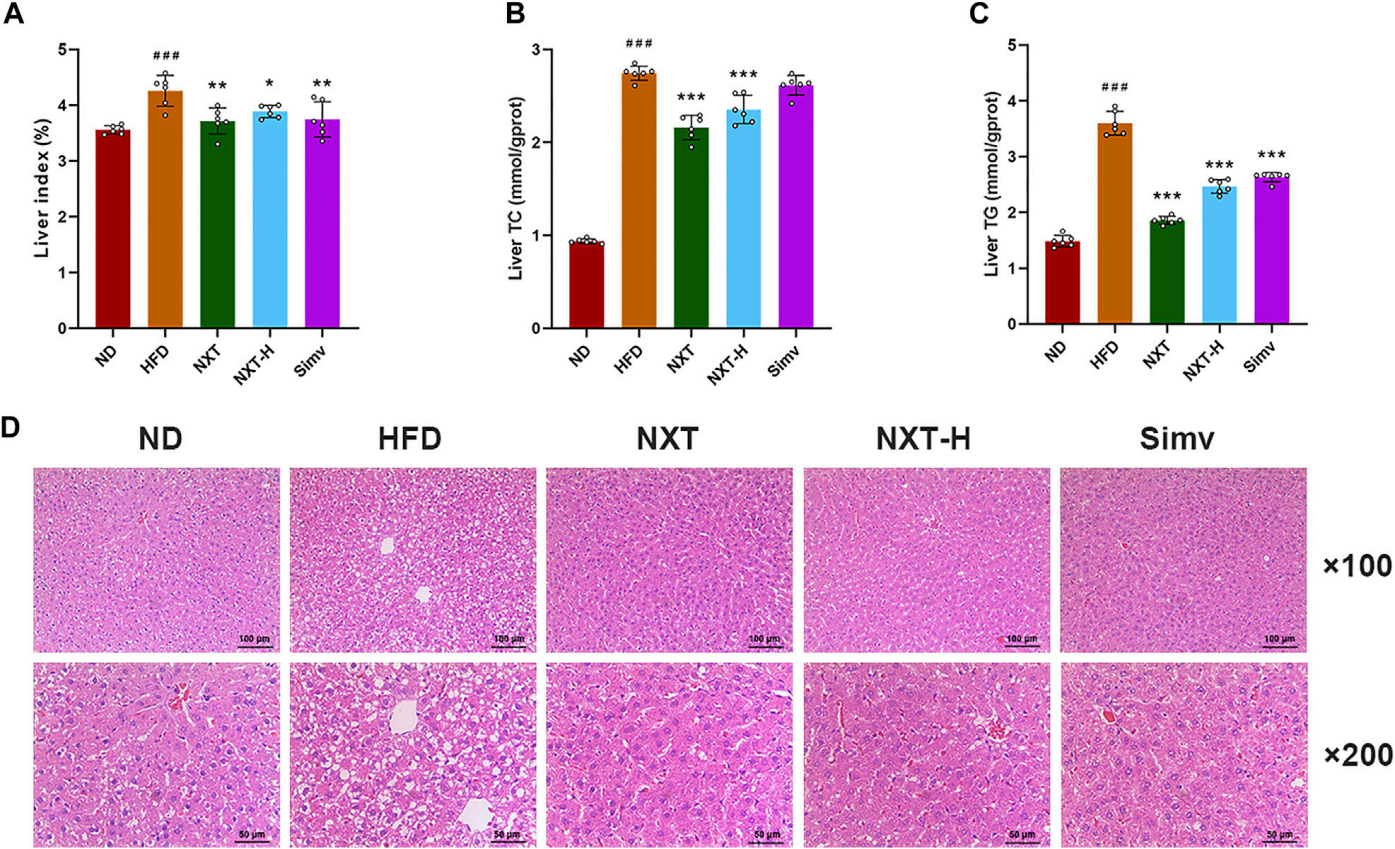
Effects of NXT on hepatic steatosis (*n* = 6 for each group). **(A–C)** Levels of liver index, liver levels of TC and TG; **(D)** H&E staining of liver tissue sections. Data are presented as means ± SD, and analyzed using the one-way ANOVA test with Dunnett method. ^###^ *p* < 0.001 compared with ND group; ^*^ *p* < 0.05, ^**^ *p* < 0.01, ^***^ *p* < 0.001 compared with HFD group.

Fat accumulation in liver is also an important indication of HFD-induced hyperlipidemia (Chen et al., 2020). Excess lipids in hepatocytes can cause lipoapoptosis and lipotoxicity, which can further lead to fatty liver disease and other liver injuries (Ou-Yang et al., 2018). Thus, the liver indexes, liver levels of TC, TG and histology of liver tissues, which are routine parameters to characterize hyperlipidemia, were measured in the present study. The liver index values of rats were increased significantly after the 6-weeks intervention (Figure 2A). The effect of HFD feeding was eliminated by co-administration of NXT (both normal and high dosage) or Simv. The levels of liver TC and TG were in the similar manner but more dramatically (Figures 2B,C). Normal dose of NXT showed slightly better liver preventive effect than NXT-H and Simv. In NXT group, the average levels of liver TC and TG decreased by 24.3% and 47.4% comparing with HFD group. H&E staining results revealed that the liver of rats in HFD group had obvious vacuolar degeneration and cell hypertrophy (Figure 2D). The degree of vacuolar degeneration in NXT, NXT-H and Simv groups was significantly reduced, indicating that NXT had potent anti-hypertrophy effect in HFD-fed rats.

Collectively, the abovementioned results indicated that NXT can reduce body weight gain, alleviate related parameters (TC, TG, HDL-C, and LDL-C) and prevent liver from HFD induced dysbiosis. Among the tested groups, NXT group exhibited the best effect (at the same level of Simv) in most parameters. At the same time, high dosage of NXT, which is twice the maximum clinical dosage, showed equal or less efficiency in alleviation of physiological status of rats. Together considering that Simv is not a positive control in maintain the balance of gut microbiota, only the samples from ND, HFD and NXT group were analyzed in the following sections.

### NXT Alters the Composition of Gut Microbiota in HFD-Fed Rats

**FIGURE 3:**
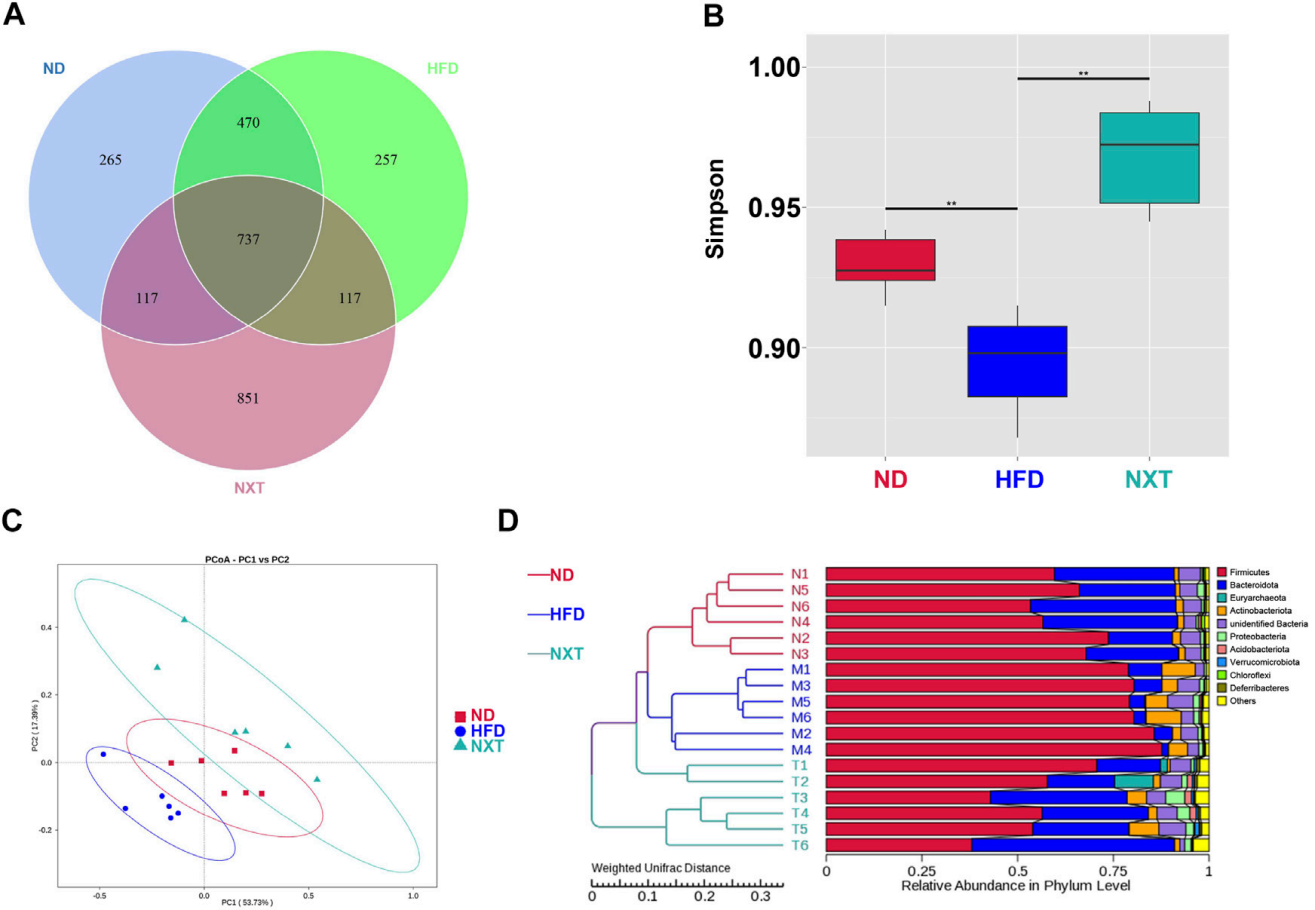
Analysis of the variation of gut microbiota among ND, HFD and NXT groups (*n* = 6 for each group). **(A)** Venn diagram; **(B)** Simpson index; **(C)** PCoA plot; **(D)** UPGMA analysis.

It was reported that some changes in the bacterial populations were associated with variations in metabolic health parameters (Patrone et al., 2016). Subsequently, the gut microbiota composition was determined by 16S rRNA sequencing (V3 + V4 region) with the Illumina MiSeq platform. A total of 1011708 raw sequences were produced in the high throughput pyrosequencing. Based on 97% identity level, the selected sequences were clustered into 951944 OTUs. Grade-abundance curves were drawn according to the abundance of OTUs classes to evaluate the species richness of the selected samples (Supplementary Figure S2A). The obtained Venn diagram showed the unique and shared OTU numbers as in Figure 3A. It was of interest that HFD did not induced significant decrease of the OTU numbers comparing with the ND group. However, the co-administration of NXT greatly enlarged the numbers of OTUs in rat feces, suggesting that the effect of NXT on hyperlipidemia in rats might be related to the species diversity of gut microbiota. The alpha diversity analysis, especially the results of Simpson as presented in Figure 3B, was in consistence with the tendency of OTU numbers. Compared with the ND group, Simpson index in the HFD group was significantly dropped and while the values of NXT group were greatly increased. PCoA (Figure 3C), as well as UPGMA clustering at phylum level (Figure 3D), revealed significantly different structural patterns among the three groups.

**FIGURE 4:**
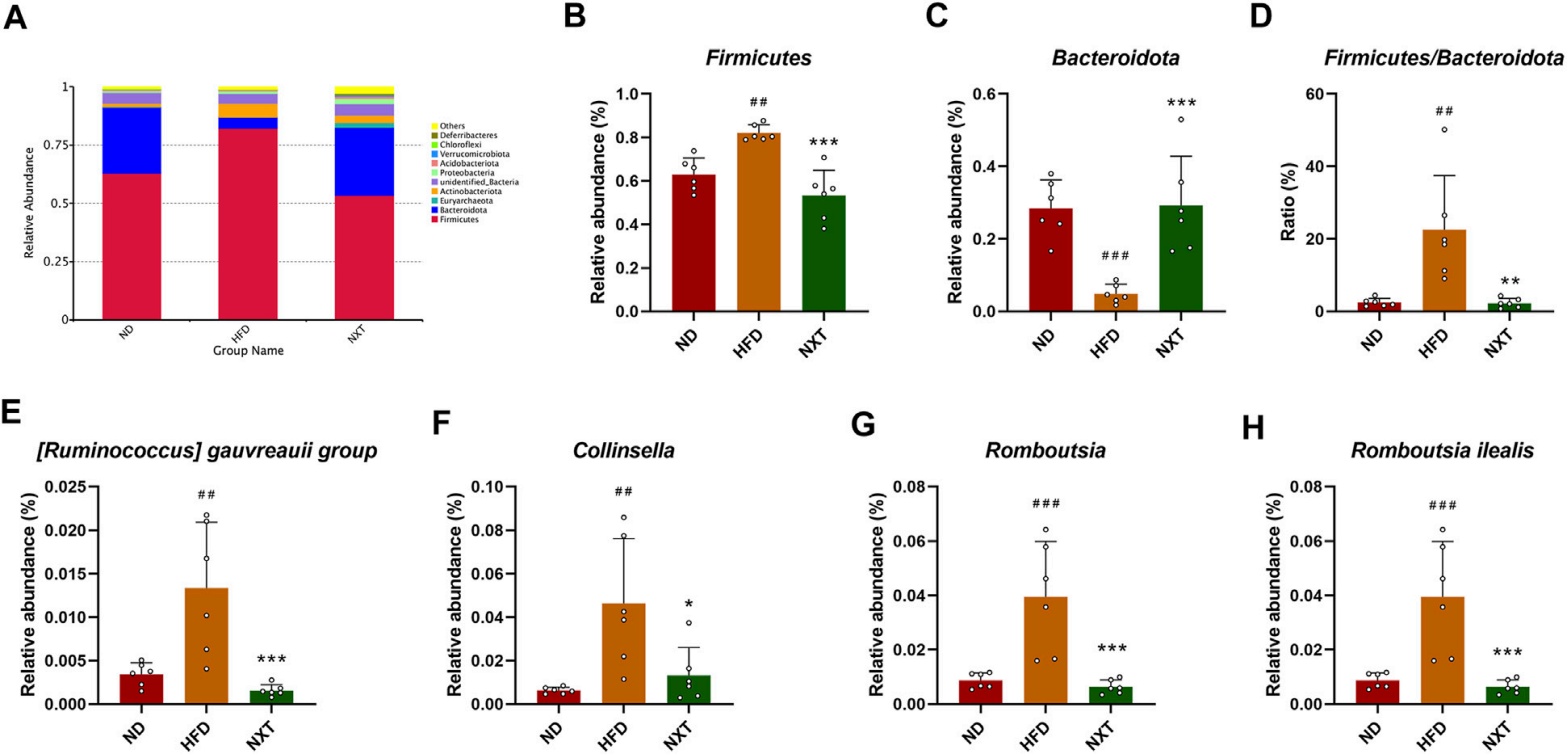
Variation of gut microbiota among ND, HFD, and NXT groups (*n* = 6 for each group) at different taxonomic levels. **(A)** Relative abundance of species at phylum level (Top 10); **(B)** Relative abundance of *Firmicute* (phylum); **(C)** Relative abundance of *Bacteroidota* (phylum); **(D)** *Firmicutes* to *Bacteroidota* ratio; **(E)** Relative abundance of [*Ruminococcus*] *gauvreauii group* (genus); **(F)** Relative abundance of *Collinsella* (genus); **(G)** Relative abundance of *Romboutsia* (genus); **(H)** Relative abundance of *Romboutsia ilealis* (species). Data are presented as means ± SD, and analyzed using the one-way ANOVA test with Dunnett method. ^##^ *p* < 0.01, ^###^ *p* < 0.001 compared with ND group; ^*^ *p* < 0.05, ^**^ *p* < 0.01, ^***^ *p* < 0.001 compared with HFD group.

The taxonomy-based investigation was also performed to reveal the effect of HFD and NXT treatment to the gut microbial community. At the phylum level, *Firmicutes* and *Bacteroidetes* were the most abundant bacteria in ND and NXT group, while in HFD group, the three biggest phyla in relative abundance were *Firmicutes*, *Actinobacteriota* and *Bacteroidetes* (Figure 4A). It is known that *Bacteroidetes* and *Firmicutes* are the dominant bacteria in human gut microbiota, accounting for more than 90% portion. As reported in recent researches, an increased ratio between the two phyla (F/B ratio) was reported to promote the development of obesity and hyperlipidemia (Magne et al., 2020). The anti-obesity and anti-hyperlipidemia activity of several components from herbal extracts, especially phenolic compounds, were reported to closely associated with their ability in down-regulating the F/B ratio of the gut microbiota (Zhu et al., 2020). In the present research, the relative abundance of *Firmicutes* in the HFD group was significantly increased (Figure 4B) while that of *Bacteroidetes* was significantly decreased (Figure 4C). This led to the great increase of the average F/B ratio to 22.5 folds in HFD group, which was 9.1 times than that of the ND group (Figure 4D). NXT administration clearly restored the F/B ratio back to the level of normal rats, suggesting that NXT has a valid capacity in alternation of gut microbial community. Considering the fact that NXT is particularly rich in phenolic compounds, these results are consistent with previous studies (Zhu et al., 2020).

In order to gain further insight of the variation of gut microbiota and its function, several representative genus/species were selected and analyzed in detail. The relative abundance of the five extracted genus/species, namely [*Ruminococcus*] *gauvreaui group*, *Collinsella*, *Romboutsia* (genus) and *Romboutsia ilealis* (species), were all up-regulated significantly by HFD treatment and brought back to low level by NXT administration (Figures 4E–H). Specifically, the fecal relative abundance of [*Ruminococcus*] *gauvreaui group* in the type 2 diabetes model rats was found increased significantly (Xu et al., 2021), while in the present research, HFD treatment also induced a 289.4% rise of relative abundance and NXT decreased the value to 88.3% of the HFD group. The relative abundance of *Collinsella*, a microorganism associated with the risk of fatty liver disease (Astbury et al., 2020), was also found in the same pattern. *Romboutsia* is also an important microorganism related to the production of acetic acid (Liu et al., 2021) and butyric acid (Qin et al., 2021). At the species level, the different treatments significantly altered the relative abundances of *Romboutsia ilealis*. This species of bacteria was reported a worsener/pathobiont in modulation of the glucose metabolism of the host (Rodrigues et al., 2021), which was in consistence with our results. Relative abundances of several other representative fecal bacteria were summarized in Supplementary Figure S3.

**FIGURE 5:**
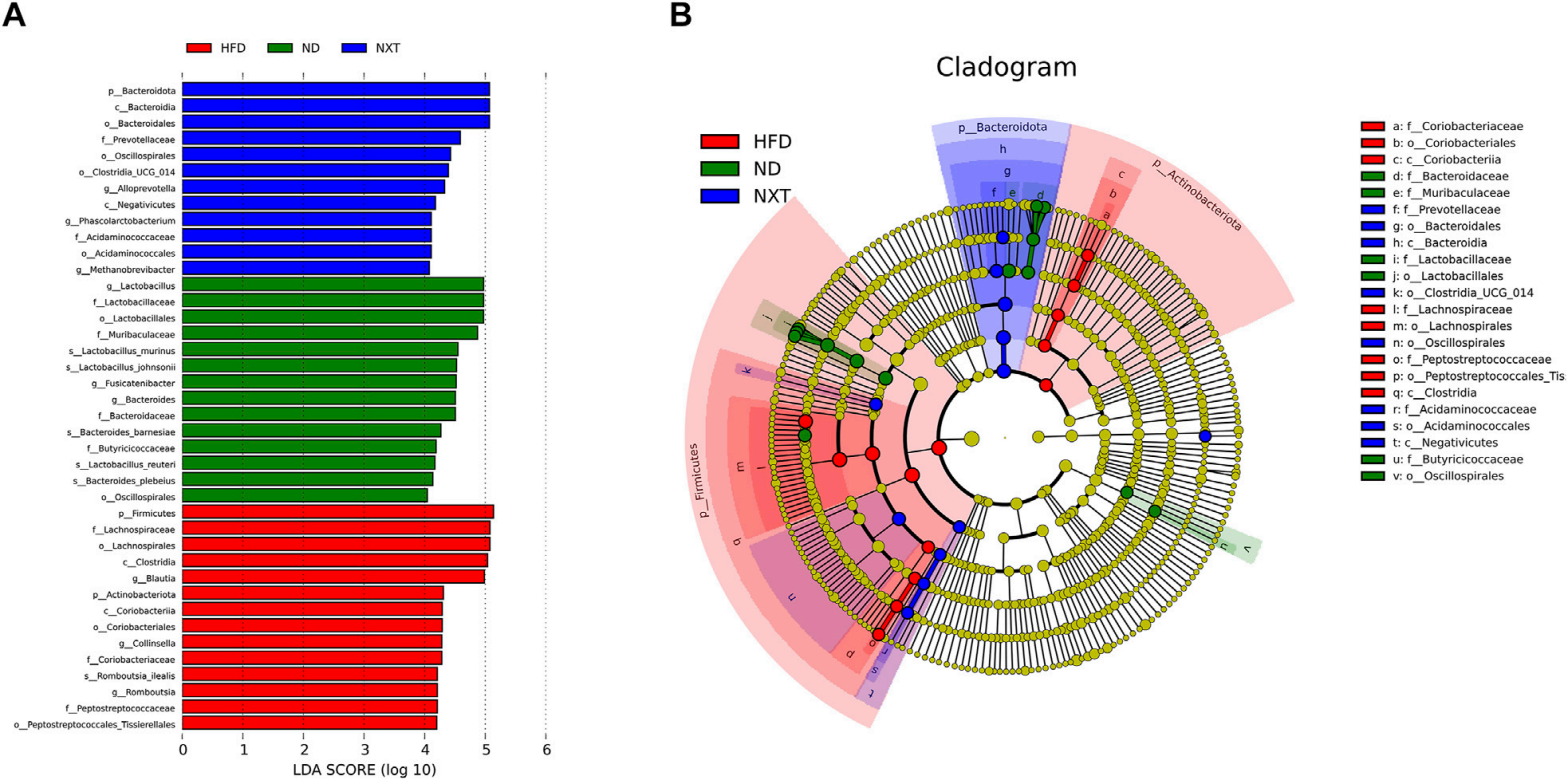
LEfSe results among the ND, HFD, and NXT group (*n* = 6 for each group). **(A)** The logarithmic LDA scores (threshold 4.0) of the dominant biomarker taxa; **(B)** Taxonomic cladogram.

In addition, LEfSe method was applied to look for the biomarkers of hyperlipidemia in gut microbiota (Figure 5). In total, there were 14, 14, and 12 significantly different OTUs in ND, HFD and NXT group, respectively (Figure 5A). The different level of labeled taxa obtained from LEfSe in the experimental group were also supported by the Cladogram analysis (Figure 5B).

### NXT Restores the Functional Pathways of Gut Microbiota

**FIGURE 6:**
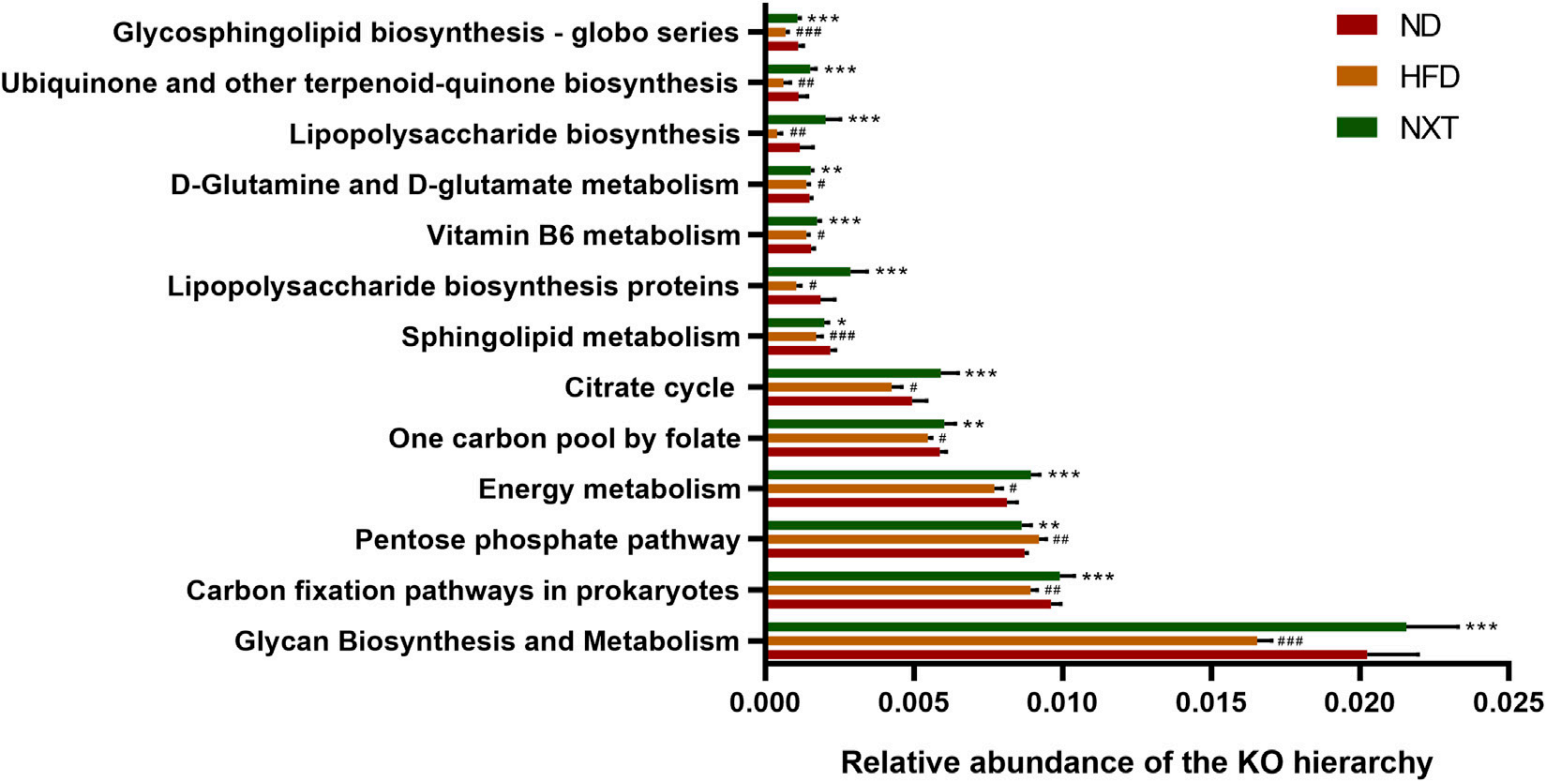
Metabolic pathways predicted by PICRUST analysis. Data are presented as means ± SD, and analyzed using the one-way ANOVA test with Dunnett method. ^#^ *p* < 0.01, ^##^ *p* < 0.01, ^###^ *p* < 0.001 compared with ND group; ^*^ *p* < 0.05, ^**^ *p* < 0.01, ^***^ *p* < 0.001 compared with HFD group.

To figure out the potential function of the gut microbiota within various groups, enriched metabolic pathways from KEGG in each group were predicted by PICRUSt. Figure 6 indicated that pentose phosphate pathway (PPP) was enhanced in HFD group, while the functional gene pathway such as glycan biosynthesis and metabolism, carbon fixation pathways in prokaryotes, energy metabolism, one carbon pool by folate, citrate cycle, sphingolipid metabolism, lipopolysaccharide biosynthesis proteins, vitamin B6 metabolism, D-glutamine and D-glutamate metabolism, lipopolysaccharide biosynthesis, ubiquinone and other terpenoid-quinone biosynthesis and glycosphingolipid biosynthesis—globo series were weakened in HFD group. These changes might lead to the accumulation of lipid in the host body and further formation of hyperlipidemia. Moreover, these affections of HFD were at least partially reversed by co-administration of NXT as shown in Figure 6. For instance, PPP is a form of oxidative decomposition of glucose that produces large amounts of NADPH and provides a reducing agent for various cellular reactions, such as the synthesis of fatty acids and sterols (Jin et al., 2018). In the present study, the enhanced PPP was down-regulated by NXT administration, which indicated that NXT might alleviate hyperlipidemia *via* inhibition of PPP.

### NXT Promotes SCFA Production in HFD-Fed Rats

**FIGURE 7:**
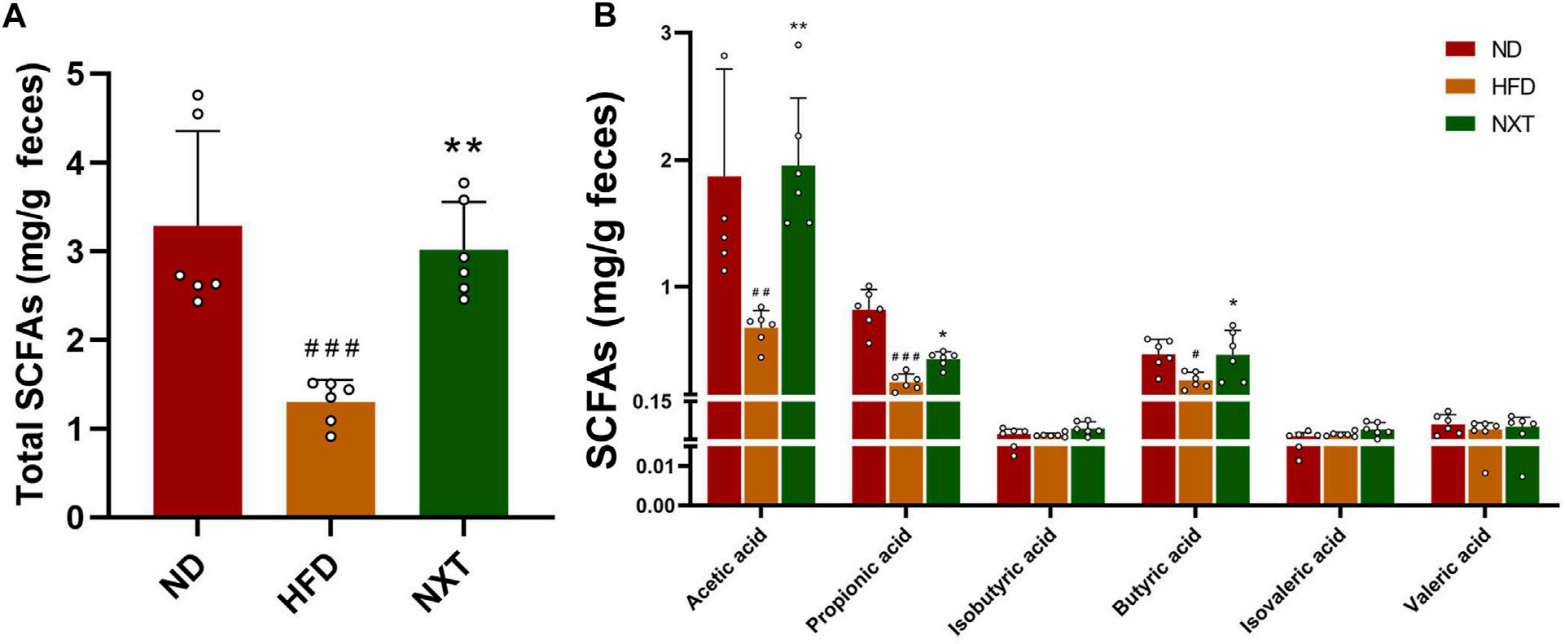
The effects of NXT on fecal concentrations of SCFAs (*n* = 6 for each group). **(A)** Total SCFAs content; **(B)** Content of acetic acid, propionic acid, isobutyric acid, butyric acid, isovaleric acid, and valeric acid content. Data are presented as means ± SD, and analyzed using the one-way ANOVA test with Dunnett method. ^##^ *p* < 0.05, ^##^ *p* < 0.01, ^###^ *p* < 0.001 compared with ND group; ^*^ *p* < 0.05, ^**^ *p* < 0.01, compared with HFD group.

Hyperlipidemia and other obesity-related dysfunctions are usually accompanied with a decrease in levels of SCFAs (Wang W. et al., 2020), such as acetic acid, propionic acid, isobutyric acid, butyric acid, isovaleric acid and valeric acid. On the contrary, specific SCFAs can inhibit hyperlipidemia by affecting energy metabolism, inflammation, and lipid oxidation. Above all, it was reported that an increase in SCFA concentration (especially acetic acid and butyric acid) improves intestinal barrier integrity, which is quite crucial to alleviate inflammation (Chang et al., 2015). As shown in Figure 7, the fecal levels of total SCFAs, acetic acid, propionic acid and butyric acid in HFD group were significantly decreased, while the levels were restored to normal after treatment with NXT (Figures 7A,B). Meanwhile, the other SCFAs, namely isobutyric acid, isovaleric acid and valeric acid, remained in the same level in all the three groups. It is known that changes in total SCFAs can regulate intestinal pH. The significant decrease in total SCFAs in HFD group can lead to an increase in intestinal pH, thus affecting intestinal homeostasis. NXT significantly improved the level of total SCFAs and reversed the disturbance in the present study. The results indicated that NXT can significantly improve the level of acetic acid, propionic acid and butyric acid, which were reported beneficial to glucose and energy homeostasis (den Besten et al., 2015). These results on SCFAs were also in consistence with previous reports on herbal phenolic compounds protecting the model animals against hyperlipidemia (Bai et al., 2020). In hence, we speculated that NXT might play an important role in the treatment of hyperlipidemia by regulating the level of intestinal SCFAs.

### NXT Modulates the Bile Acid Profiles in Gut

**FIGURE 8:**
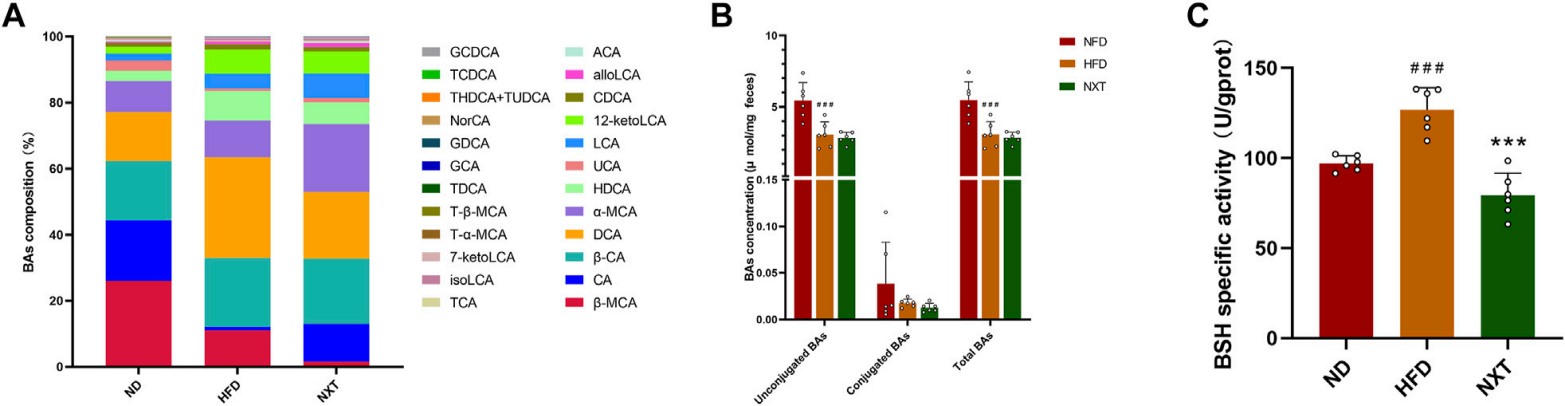
The effects of NXT on BA metabolism (*n* = 6 for each group). **(A)** The relative composition of BAs; **(B)** The concentrations of unconjugated, conjugated and total BAs in rat feces; **(C)** The BSH specific activities. Data are presented as means ± SD, and analyzed using the one-way ANOVA test with Dunnett method. ^##^ *p* < 0.01, ^###^ *p* < 0.001 compared with ND group; ^**^ *p* < 0.01, ^***^ *p* < 0.001 compared with HFD group. alloLCA, allolithocholic acid; LCA, lithocholic acid; isoLCA, isolithocholic acid; 12-ketoLCA, 12-ketolithocholic acid; 7-ketoLCA, 7-ketolithocholic acid; DCA, deoxycholic acid; CDCA, chenodeoxycholic acid; HDCA, hyodeoxycholic acid; NorCA, norcholic acid; α-MCA, α-muricholic acid; UCA, ursocholic acid; β-MCA, β-muricholic acid; CA, cholic acid; ACA, allocholic acid; β-CA, β-cholic acid; GCDCA, glycochenodeoxycholic acid; GDCA, glycodeoxycholic acid; GCA, glycocholic acid; THDCA, taurohyodeoxycholic acid; TUDCA, tauroursodeoxycholic acid; TDCA, taurodeoxycholic acid; TCDCA, taurochenodeoxycholic acid; TCA, taurocholic acid; T-α-MCA, tauro-α-muricholic acid; T-β-MCA, tauro-β-muricholic acid.

Bile acids play pleiotropic roles in lipid metabolism and are crucial factors in the enterohepatic circulation (Clifford et al., 2021). BAs are firstly synthesized in hepatocytes from cholesterol and further modified by diverse enzymes excreted by gut microbiomes. Dysregulation of BAs has been reported to be closely associated with a lot of metabolic diseases including hyperlipidemia and atherosclerosis (Tang et al., 2019). Thus, in order to investigate the changes of fecal BA profiles induced by HFD and NXT intervention, the fecal BA profiles in ND, HFD, and NXT groups were analyzed. With the help of quantitative HPLC-MS method, 24 kinds of BAs in feces were determined. The BA profiles of the three groups were presented in Figure 8A. Further statistical analysis revealed that the fecal levels of unconjugated BAs and total BAs were significantly reduced in the HFD group compared with the ND group (Figure 8B). However, the data of NXT group was not significantly different from that of HFD group.

Various gut microbiota-derived enzymes are involved in the modification of BAs (Gu et al., 2017). For instance, 7α-dehydroxylase is the key enzyme in transformation of CA and CDCA to their lipophilic secondary BAs, DCA and LCA (Han et al., 2015). BSH is another important enzyme which can cleave the amide bond between the glycine and taurine moiety conjugated to the steroid nucleus of bile salts. This reaction leads to the deconjugation of conjugated bile acids and is the primer step before the bile acid alternation by gut microbiota (Wei et al., 2020). Herein in this study, the specific activity of BSH in the HFD group was significantly upregulated, and supplementation of NXT intensely reduced the BSH activities by 37.4% (Figure 8C). Normally, lowering of BSH activity could lead to suppressed BA deconjugation in the small intestine, which can upregulate the levels of conjugated BAs in turn. But in the present study, the concentrations of conjugated BAs were particularly low (< 0.5%) comparing with unconjugated BAs in feces. The low concentrations of conjugated BAs and individual differences among the rats might hindered the correlation, which may imply that further investigations on BA metabolism should be carried out with more precise detection and larger sample size.

### Correlation Analysis of Gut Microbiota Changes With Clinical/Metabolomic Parameters Revealed the Potential Biomarkers of NXT Treatment on Hyperlipidemia

**FIGURE 9:**
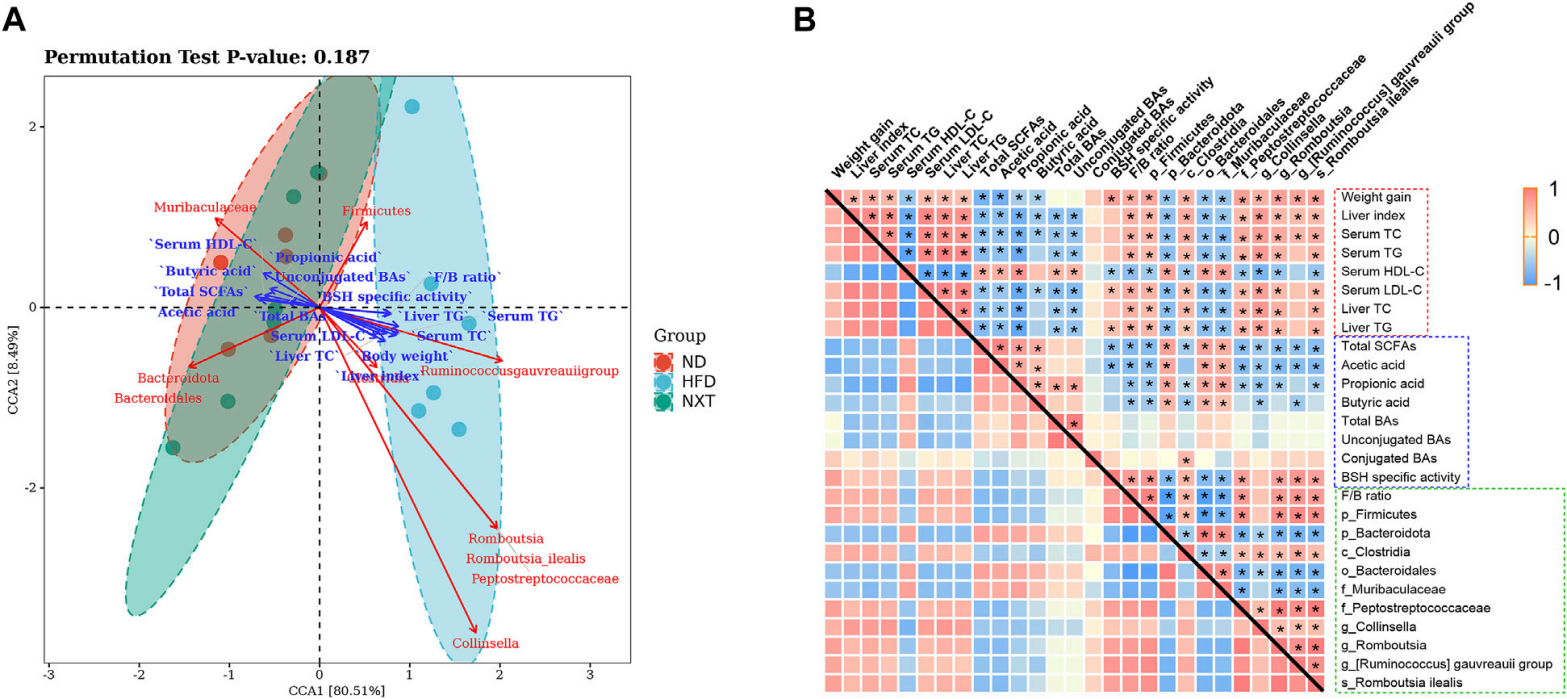
The correlation of gut microbiota changes with hyperlipidemia-related clinic or metabolomic parameters. **(A)** CCA represented the relationship among intestinal microbiota, intestinal metabolites and the clinical parameters, with acute angle indicating positive correlation and obtuse angle indicating negatively correlation; **(B)** Spearman’s correlation values were used for the matrix, with red indicating a positive correlation and blue indicating a negative correlation ^*^ *p* < 0.05.

In order to comprehensively analyze the relationship between hyperlipidemia characteristic indexes and gut microbiota, canonical correlation analysis (CCA) and Spearman’s correlation analysis were performed (Figure 9). In this study, 10 kinds of intestinal bacteria which significantly changed after supplementation of NXT and hyperlipidemia-related clinic or metabolomic parameters were employed in the correlation analysis.

As shown in Figure 9A, through CCA, we found that the 10 kinds of intestinal bacteria had different aggregations. The ND group and the NXT group were closer to each other, while the HFD group was far away from the two groups, indicating that NXT can improve the relative abundances of these intestinal bacteria. At the same time, the relative abundances of *Firmicutes*, *Clostridia*, *Collinsella*, *Romboutsia*, *Peptostreptococcaceae*, *Romboutsia ilealis* and [*Ruminococcus*] *gauvreauii* group were positively correlated with the clinical parameters but negatively associated with SCFAs and BAs. In the contrast, the relative abundances of *Bacteroidota*, *Muribaculaceae* and *Bacteroidales* were negatively associated with the clinical parameters but positively correlated with SCFAs and BAs. These results suggest that intestinal microbiota may influence intestinal metabolites and the clinical parameters of hyperlipidemia.

Spearman correlation analysis was then employed for further mining of the relationship among clinical parameters, gut microbiota and related metabolites. As shown in Figure 9B, among the gut microbiota factors, F/B ratio was found as a significant indicator closely related to the clinical parameters of hyperlipidemia. In addition, the relative abundances of *Firmicutes*, *Clostridia*, *Collinsella*, *Romboutsia*, *Peptostreptococcaceae*, *Romboutsia ilealis* and [*Ruminococcus*] *gauvreauii group* were positively correlated with the clinical parameters except serum HDL-C. In the contrast, the relative abundances of *Bacteroidota*, *Muribaculaceae* and *Bacteroidales* were negatively associated with these clinical parameters. The fecal levels of total SCFAs, acetic acid, propionic acid and butyric acid were significantly associated with clinical parameters of hyperlipidemia negatively. The fecal level of total BAs and unconjugated BAs were also significantly associated with clinical parameters negatively.

The levels of SCFAs and gut microbiota were also found interrelated. Especially, *Bacteroidota*, *Muribaculaceae* and *Bacteroidales* were found positively correlated with the fecal concentrations of total SCFAs, acetic acid, propionic acid and butyric acid.

Some correlations between gut microbiota and BAs were also revealed. For instance, the relative abundance of *Clostridia* was significantly correlated with the levels of conjugated BAs and BSH specific activity positively. The results showed that gut microbiota not only affected the metabolism of SCFAs and BAs, but also affected the host phenotypes such as serum lipid indexes and liver function parameters remotely. Administration of NXT can regulate the composition of gut microbiota community and metabolites (especially SCFAs), thus restore a relatively healthy intestinal microenvironment to prevent the occurrence and development of hyperlipidemia. In particular, several indicators of the fecal samples, including the amount of propionic aicd, *F*/*B* ratio and the relative abundance of *Collinsella* in feces, are supposed to be potential therapeutic biomarkers of NXT in the treatment of hyperlipidemia.

## Conclusion

In conclusion, the present investigation demonstrated that NXT was effective in regulation of gut microbiota and further prevention of hyperlipidemia in HFD fed rats. Supplementation of NXT can alleviate the hyperlipidemia related phenotypes including reduction of body weight, amelioration of serum lipid levels and liver function parameters. Especially, NXT greatly reversed the upregulation of HFD to liver index, liver TC and liver TG. Meanwhile, NXT also reconstituted the gut microbiota composition. Some important factors as F/B ratio, the amount of propionic aicd and the relative abundance of *Collinsella* were proposed to be potential therapeutic biomarkers. The metabolic function of gut microbiota, including SCFA levels, BA levels and BSH activities were all altered. The correlation between the gut microbiota and clinical/metabolomic parameters was also investigated. The present work might provide novel insights into the anti-hyperlipidemia effect of NXT and afford new scientific evidence for clinical application of NXT.

## Data Availability

The datasets presented in this study can be found in online repositories. The name of the repository and accession number can be found below: National Center for Biotechnology Information (NCBI) BioProject, https://www.ncbi.nlm.nih.gov/bioproject/, PRJNA795283.

## References

[B1] Astbury S. Atallah E. Vijay A. Aithal G. P. Grove J. I. Valdes A. M. (2020). Lower Gut Microbiome Diversity and Higher Abundance of Proinflammatory Genus *Collinsella* Are Associated with Biopsy-Proven Nonalcoholic Steatohepatitis. Gut Microbes 11, 569–580. 10.1080/19490976.2019.1681861 31696774 PMC7524262

[B2] Bäckhed F. Ding H. Wang T. Hooper L. V. Koh G. Y. Nagy A. (2004). The Gut Microbiota as an Environmental Factor that Regulates Fat Storage. Proc. Natl. Acad. Sci. U. S. A. 101, 15718–15723. 10.1073/pnas.0407076101 15505215 PMC524219

[B3] Bäckhed F. Ley R. E. Sonnenburg J. L. Peterson D. A. Gordon J. I. (2005). Host-bacterial Mutualism in the Human Intestine. Science 307, 1915–1920. 10.1126/science.1104816 15790844

[B4] Bai L. Gao M. Cheng X. Kang G. Cao X. Huang H. (2020). Engineered Butyrate-Producing Bacteria Prevents High Fat Diet-Induced Obesity in Mice. Microb. Cel Fact. 19, 94. 10.1186/s12934-020-01350-z PMC7183672 32334588

[B5] Chalasani N. Younossi Z. Lavine J. E. Charlton M. Cusi K. Rinella M. (2018). The Diagnosis and Management of Nonalcoholic Fatty Liver Disease: Practice Guidance from the American Association for the Study of Liver Diseases. Hepatology 67, 328–357. 10.1002/hep.29367 28714183

[B6] Chang C. J. Lin C. S. Lu C. C. Martel J. Ko Y. F. Ojcius D. M. (2015). Ganoderma Lucidum Reduces Obesity in Mice by Modulating the Composition of the Gut Microbiota. Nat. Commun. 6, 7489. 10.1038/ncomms8489 26102296 PMC4557287

[B7] Chen M. Hou P. Zhou M. Ren Q. Wang X. Huang L. (2020). Resveratrol Attenuates High-Fat Diet-Induced Non-alcoholic Steatohepatitis by Maintaining Gut Barrier Integrity and Inhibiting Gut Inflammation through Regulation of the Endocannabinoid System. Clin. Nutr. 39, 1264–1275. 10.1016/j.clnu.2019.05.020 31189495

[B8] Chen X. D'Souza R. Hong S. T. (2013). The Role of Gut Microbiota in the Gut-Brain axis: Current Challenges and Perspectives. Protein Cell 4, 403–414. 10.1007/s13238-013-3017-x 23686721 PMC4875553

[B9] Clifford B. L. Sedgeman L. R. Williams K. J. Morand P. Cheng A. Jarrett K. E. (2021). FXR Activation Protects against NAFLD via Bile-acid-dependent Reductions in Lipid Absorption. Cell Metab 33, 1671–e4. e4. 10.1016/j.cmet.2021.06.012 34270928 PMC8353952

[B10] den Besten G. Bleeker A. Gerding A. van Eunen K. Havinga R. van Dijk T. H. (2015). Short-Chain Fatty Acids Protect Against High-Fat Diet-Induced Obesity via a PPARγ-Dependent Switch From Lipogenesis to Fat Oxidation. Diabetes. 64, 2398–2408. 10.2337/db14-1213 25695945

[B11] Feng K. Zhu X. Chen T. Peng B. Lu M. Zheng H. (2019). Prevention of Obesity and Hyperlipidemia by Heptamethoxyflavone in High-Fat Diet-Induced Rats. J. Agric. Food Chem. 67, 2476–2489. 10.1021/acs.jafc.8b05632 30740980

[B12] Gu Y. Wang X. Li J. Zhang Y. Zhong H. Liu R. (2017). Analyses of Gut Microbiota and Plasma Bile Acids Enable Stratification of Patients for Antidiabetic Treatment. Nat. Commun. 8, 1785. 10.1038/s41467-017-01682-2 29176714 PMC5702614

[B14] Han J. Liu Y. Wang R. Yang J. Ling V. Borchers C. H. (2015). Metabolic Profiling of Bile Acids in Human and Mouse Blood by LC-MS/MS in Combination with Phospholipid-Depletion Solid-phase Extraction. Anal. Chem. 87, 1127–1136. 10.1021/ac503816u 25496250

[B15] Huang Z. R. Deng J. C. Li Q. Y. Cao Y. J. Lin Y. C. Bai W. D. (2020). Protective Mechanism of Common Buckwheat (Fagopyrum Esculentum Moench.) against Nonalcoholic Fatty Liver Disease Associated with Dyslipidemia in Mice Fed a High-Fat and High-Cholesterol Diet. J. Agric. Food Chem. 68, 6530–6543. 10.1021/acs.jafc.9b08211 32383865

[B16] Jia X. Xu W. Zhang L. Li X. Wang R. Wu S. (2021). Impact of Gut Microbiota and Microbiota-Related Metabolites on Hyperlipidemia. Front. Cel. Infect. Microbiol. 11, 634780. 10.3389/fcimb.2021.634780 PMC8417472 34490132

[B17] Jin E. S. Lee M. H. Murphy R. E. Malloy C. R. (2018). Pentose Phosphate Pathway Activity Parallels Lipogenesis but Not Antioxidant Processes in Rat Liver. Am. J. Physiol. Endocrinol. Metab. 314, E543–E551. 10.1152/ajpendo.00342.2017 29351478 PMC6032064

[B18] Liang Q. Cai Y. Chen R. Chen W. Chen L. Xiao Y. (2018). The Effect of Naoxintong Capsule in the Treatment of Patients with Cerebral Infarction and Carotid Atherosclerosis: a Systematic Review and Meta-Analysis of Randomized Trials. Evid. Based Complement. Alternat Med. 2018, 5892306. 10.1155/2018/5892306 30140296 PMC6081578

[B19] Liu Y. Xue K. Iversen K. N. Qu Z. Dong C. Jin T. (2021). The Effects of Fermented rye Products on Gut Microbiota and Their Association with Metabolic Factors in Chinese Adults - an Explorative Study. Food Funct. 12, 9141–9150. 10.1039/d1fo01423d 34397057

[B20] Magne F. Gotteland M. Gauthier L. Zazueta A. Pesoa S. Navarrete P. (2020). The *Firmicutes/Bacteroidetes* Ratio: A Relevant Marker of Gut Dysbiosis in Obese Patients? Nutrients 12, 1474. 10.3390/nu12051474 PMC7285218 32438689

[B42] Opoku S. Gan Y. Fu W. Chen D. Addo-Yobo E. Trofimovitch D. (2019). Prevalence and Risk Factors for Dyslipidemia Among Adults in Rural and Urban China: Findings From the China National Stroke Screening and Prevention Project (CNSSPP) BMC Public Health. 19 (1), 1500. 10.1186/s12889-019-7827-5 31711454 PMC6849283

[B21] Ou-Yang Q. Xuan C. X. Wang X. Luo H. Q. Liu J. E. Wang L. L. (2018). 3-Acetyl-oleanolic Acid Ameliorates Non-alcoholic Fatty Liver Disease in High Fat Diet-Treated Rats by Activating AMPK-Related Pathways. Acta Pharmacol. Sin. 39, 1284–1293. 10.1038/aps.2017.142 29345253 PMC6289400

[B22] Patrone V. Vajana E. Minuti A. Callegari M. L. Federico A. Loguercio C. (2016). Postoperative Changes in Fecal Bacterial Communities and Fermentation Products in Obese Patients Undergoing Bilio-Intestinal Bypass. Front. Microbiol. 7, 200. 10.3389/fmicb.2016.00200 26941724 PMC4762995

[B23] Qin R. Wang J. Chao C. Yu J. Copeland L. Wang S. (2021). RS5 Produced More Butyric Acid through Regulating the Microbial Community of Human Gut Microbiota. J. Agric. Food Chem. 69, 3209–3218. 10.1021/acs.jafc.0c08187 33630575

[B24] Rodrigues R. R. Gurung M. Li Z. García-Jaramillo M. Greer R. Gaulke C. (2021). Transkingdom Interactions between Lactobacilli and Hepatic Mitochondria Attenuate Western Diet-Induced Diabetes. Nat. Commun. 12, 101. 10.1038/s41467-020-20313-x 33397942 PMC7782853

[B25] Shi Z. Zhu Y. Teng C. Yao Y. Ren G. Richel A. (2020). Anti-obesity Effects of α-amylase Inhibitor Enriched-Extract from white Common Beans (Phaseolus vulgaris L.) Associated with the Modulation of Gut Microbiota Composition in High-Fat Diet-Induced Obese Rats. Food Funct. 11, 1624–1634. 10.1039/c9fo01813a 32022058

[B26] Shin N. R. Lee J. C. Lee H. Y. Kim M. S. Whon T. W. Lee M. S. (2014). An Increase in the *Akkermansia* Spp. Population Induced by Metformin Treatment Improves Glucose Homeostasis in Diet-Induced Obese Mice. Gut 63, 727–735. 10.1136/gutjnl-2012-303839 23804561

[B28] Tang W. H. W. Li D. Y. Hazen S. L. (2019). Dietary Metabolism, the Gut Microbiome, and Heart Failure. Nat. Rev. Cardiol. 16, 137–154. 10.1038/s41569-018-0108-7 30410105 PMC6377322

[B29] Tong X. Xu J. Lian F. Yu X. Zhao Y. Xu L. (2018). Structural Alteration of Gut Microbiota during the Amelioration of Human Type 2 Diabetes with Hyperlipidemia by Metformin and a Traditional Chinese Herbal Formula: A Multicenter, Randomized, Open Label Clinical Trial. MBio 9, e02392–17. 10.1128/mBio.02392-17 29789365 PMC5964358

[B30] Wang H. Y. Zhou H. F. He Y. Yu L. Li C. Yang J. H. (2020a). Protective Effect of Naoxintong Capsule () Combined with Guhong Injection () on Rat Brain Microvascular Endothelial Cells during Cerebral Ischemia-Reperfusion Injury. Chin. J. Integr. Med. 27, 744. 10.1007/s11655-020-3215-3 32248514

[B43] Wang S. Xu H. Ma Y. Wang X. Shi Y. Huang B. (2015). Characterization and Rapid Identification of Chemical Constituents of NaoXinTong Capsules by UHPLC-linear ion Trap/Orbitrap Mass Spectrometry. J. Pharm. Biomed. Anal. 111, 104–118. 10.1016/j.jpba.2015.01.020 25880241

[B31] Wang W. Zhong M. Yu T. Chen L. Shi L. Zong J. (2020c). Polysaccharide Extracted from WuGuChong Reduces High-Fat Diet-Induced Obesity in Mice by Regulating the Composition of Intestinal Microbiota. Nutr. Metab. (Lond) 17, 27. 10.1186/s12986-020-00442-2 32256675 PMC7106597

[B32] Wang Y. Yan X. Mi S. Li Z. Wang Y. Zhu H. (2017). Naoxintong Attenuates Ischaemia/reperfusion Injury through Inhibiting NLRP3 Inflammasome Activation. J. Cel. Mol. Med. 21, 4–12. 10.1111/jcmm.12915 PMC5192872 27785882

[B33] Wang Y. Yao W. Li B. Qian S. Wei B. Gong S. (2020b). Nuciferine Modulates the Gut Microbiota and Prevents Obesity in High-Fat Diet-Fed Rats. Exp. Mol. Med. 52, 1959–1975. 10.1038/s12276-020-00534-2 33262480 PMC8080667

[B34] Wei M. Huang F. Zhao L. Zhang Y. Yang W. Wang S. (2020). A Dysregulated Bile Acid-Gut Microbiota axis Contributes to Obesity Susceptibility. EBioMedicine 55, 102766. 10.1016/j.ebiom.2020.102766 32408110 PMC7225614

[B35] Wu C. Xi C. Tong J. Zhao J. Jiang H. Wang J. (2019). Design, Synthesis, and Biological Evaluation of Novel Tetrahydroprotoberberine Derivatives (THPBs) as Proprotein Convertase Subtilisin/kexin Type 9 (PCSK9) Modulators for the Treatment of Hyperlipidemia. Acta Pharm. Sin. B 9, 1216–1230. 10.1016/j.apsb.2019.06.006 31867167 PMC6900552

[B36] Xu T. Ge Y. Du H. Li Q. Xu X. Yi H. (2021). Berberis Kansuensis Extract Alleviates Type 2 Diabetes in Rats by Regulating Gut Microbiota Composition. J. Ethnopharmacol. 273, 113995. 10.1016/j.jep.2021.113995 33675912

[B44] Xu H. Shi Y. Zhang Y. Jia Q. Li D. Zhang Y. (2016). Identification of Key Active Constituents of Buchang Naoxintong Capsules With Therapeutic Effects Against Ischemic Stroke by Using an Integrative Pharmacology-Based Approach. Mol. Biosyst. 12, 233–245. 10.1039/c5mb00460h 26588440

[B37] Xue J. Zhang X. Zhang C. Kang N. Liu X. Yu J. (2016). Protective Effect of Naoxintong against Cerebral Ischemia Reperfusion Injury in Mice. J. Ethnopharmacol. 182, 181–189. 10.1016/j.jep.2016.02.022 26902830

[B38] Yan Z. Wu H. Zhou H. Chen S. He Y. Zhang W. (2020). Integrated Metabolomics and Gut Microbiome to the Effects and Mechanisms of Naoxintong Capsule on Type 2 Diabetes in Rats. Sci. Rep. 10, 10829. 10.1038/s41598-020-67362-2 32616735 PMC7331749

[B39] Zhang F. Huang B. Zhao Y. Tang S. Xu H. Wang L. (2013). BNC Protects H9c2 Cardiomyoblasts from H_2_O_2_ -Induced Oxidative Injury through ERK1/2 Signaling Pathway. Evid. Based Complement. Alternat Med. 2013, 802784. 10.1155/2013/802784 24223618 PMC3810482

[B40] Zhang W. J. Su W. W. Li P. B. Rao H. Y. Lin Q. W. Zeng X. (2019). Naoxintong Capsule Inhibits the Development of Cardiovascular Pathological Changes in Bama Minipig through Improving Gut Microbiota. Front. Pharmacol. 10, 1128. 10.3389/fphar.2019.01128 31632272 PMC6785636

[B41] Zhu Y. Zhang J. Y. Wei Y. L. Hao J. Y. Lei Y. Q. Zhao W. B. (2020). The Polyphenol-Rich Extract from Chokeberry (Aronia Melanocarpa L.) Modulates Gut Microbiota and Improves Lipid Metabolism in Diet-Induced Obese Rats. Nutr. Metab. (Lond) 17, 54. 10.1186/s12986-020-00473-9 32655675 PMC7339576

