# Supplementary material for: Naoxintong Capsule Alternates Gut Microbiota and Prevents Hyperlipidemia in High-Fat-Diet Fed Rats
Source: Front Pharmacol. 2022 Mar 21;13:843409. doi: 10.3389/fphar.2022.843409 (PMC8978017; doi:10.3389/fphar.2022.843409)
Supplement: Supplementary file 1 [file DataSheet1.DOCX]

Supplementary Material

# Supplementary Table S1. Mass spectrometry data of the major components in NXT

| Peak No. | t_R_ (min) | Measured mass | Molecular formula | Identification | Error (ppm) |
| --- | --- | --- | --- | --- | --- |
| 1 | 8.25 | 243.0623 | C_9_H_12_N_2_O_6_ | uridine | 4.515 |
| 2 | 12.08 | 282.0844 | C_10_H_13_N_5_O_5_ | guanosine | 3.953 |
| 3 | 14.03 | 267.0734 | C_10_H_12_N_4_O_6_ | inosine | 3.722 |
| 4 | 41.96 | 289.0719 | C_15_H_14_O_6_ | catechin | 4.377 |
| 5 | 43.60 | 353.0880 | C_16_H_18_O_9_ | chlorogenic acid | 3.686 |
| 6 | 55.02 | 525.1613 | C_24_H_30_O_13_ [M+COOH]^-^ | paeoniflorin | 1.871 |
| 7 | 55.33 | 611.1620 | C_27_H_32_O_16_ | hydroxysafflor yellow A | 2.273 |
| 8 | 66.99 | 631.1675 | C_30_H_32_O_15_ | galloylpaeoniflorin | 2.746 |
| 9 | 74.41 | 537.1044 | C_27_H_22_O_12_ | lithospermic acid | 3.123 |
| 10 | 78.27 | 1043.2692 | C_48_H_52_O_26_ | Anhydrosafflor yellow B | 2.744 |
| 11 | 79.61 | 359.0778 | C_18_H_16_O_8_ | rosmarinic acid | 4.501 |
| 12 | 82.72 | 493.1147 | C_26_H_22_O_10_ | salvianolic acid A | 3.441 |
| 13 | 87.20 | 717.1467 | C_36_H_30_O_16_ | salvianolic acid B | 2.397 |
| 14 | 88.79 | 283.0614 | C_16_H_12_O_5_ | calycosin | 4.487 |

# Supplementary Table S2. Mass spectrometry factors of bile acids detected

| No. | Bile acid | CAS No. | Mother ion | Sub ion |
| --- | --- | --- | --- | --- |
| 1 | alloLCA | 2276-93-9 | 375.145 | 375.145 |
| 2 | LCA | 434-13-9 | 375.3 | 375.3 |
| 3 | isoLCA | 1534-35-6 | 375.301 | 375.301 |
| 4 | 12-ketoLCA | 5130-29-0 | 389.314 | 389.314 |
| 5 | 7-ketoLCA | 4651-67-6 | 389.311 | 389.311 |
| 6 | DCA | 83-44-3 | 391.3 | 391.3 |
| 7 | CDCA | 474-25-9 | 391.301 | 391.301 |
| 8 | HDCA | 83-49-8 | 391.303 | 391.303 |
| 9 | NorCA | 60692-62-0 | 393.211 | 329.1 |
| 10 | α-MCA | 2393-58-0 | 407.3 | 407.3 |
| 11 | UCA | 2955-27-3 | 407.301 | 407.301 |
| 12 | β-MCA | 2393-59-1 | 407.302 | 407.302 |
| 13 | CA | 81-25-4 | 407.303 | 407.303 |
| 14 | ACA | 2464-18-8 | 407.304 | 407.304 |
| 15 | β-CA | 3338-16-7 | 407.362 | 407.362 |
| 16 | GCDCA | 16564-43-5 | 448.276 | 73.9 |
| 17 | GDCA | 16409-34-0 | 448.279 | 73.9 |
| 18 | GCA | 863-57-0 | 464.281 | 73.9 |
| 19 | THDCA  +TUDCA | 38411-85-7  35807-85-3 | 498.224 | 79.9 |
| 20 | TDCA | 1180-95-6 | 498.35 | 79.8 |
| 21 | TCDCA | 6009-98-9 | 498.357 | 79.8 |
| 22 | TCA | 145-42-6 | 514.332 | 79.8 |
| 23 | T-α-MCA | 25696-60-0 | 514.337 | 79.9 |
| 24 | T-β-MCA | 145022-92-0 | 514.346 | 79.9 |

# Supplementary Figures


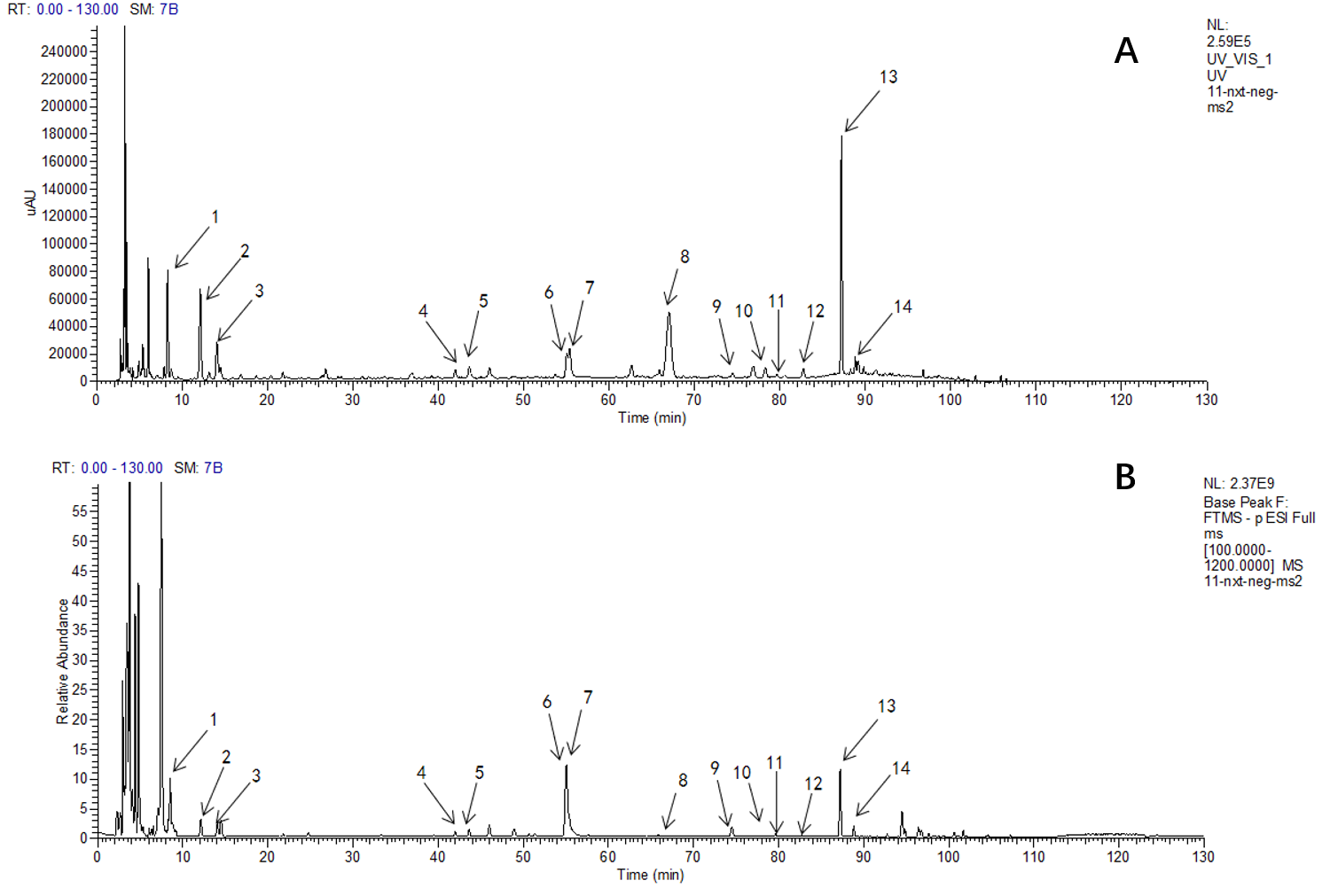


**Supplementary Figure S1.** (A) UV (wavelength=254 nm) and (B) Base Peak Chromatogram of NXT. **1**-uridine, **2**-guanosine, **3**-inosine, **4**-catechin, **5**-chlorogenic acid, **6**-paeoniflorin, **7**-hydroxysafflor yellow A, **8**-galloylpaeoniflorin, **9**-lithospermic acid, **10**-anhydrosafflor yellow B, **11**-rosmarinic acid, **12**-salvianolic acid A, **13**-salvianolic acid B, **14**-calycosin.


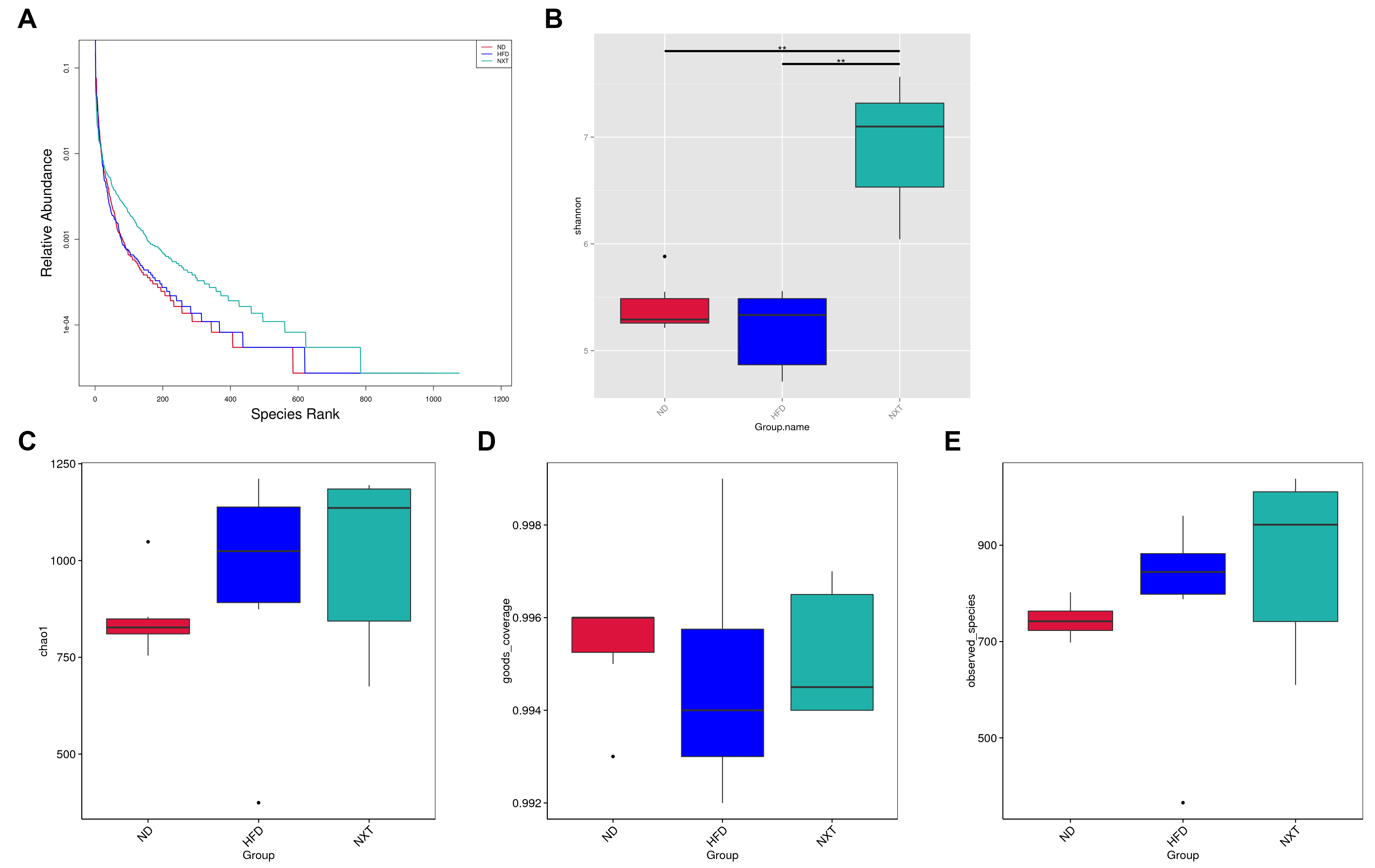


**Supplementary Figure S2.** Alpha diversity in three groups (n=6 for each group). (A) Rank-abundance (B) Shannon (C) Chao1 (D) Goods_coverage (E) Observed_species.


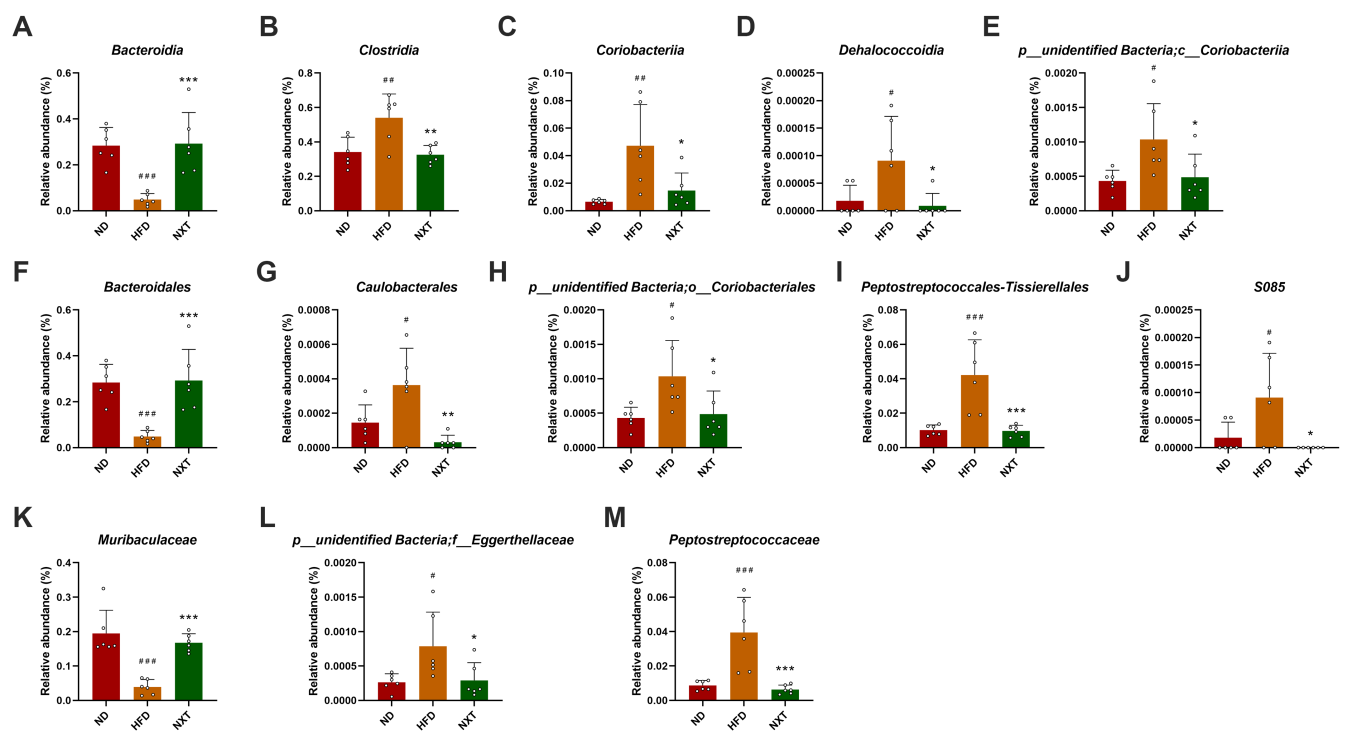


**Supplementary Figure S3.** Relative abundance of fecal bacteria of rats in three groups (n=6 for each group). (A) *Bacteroidia* (B) *Clostridia* (C) *Coriobacteriia* (D) *Dehalococcoidia* (E) *p__unidentified_Bacteria;c__Coriobacteriia* (F) *Bacteroidales* (G) *Caulobacterales* (H) *p__unidentified_Bacteria;o__Coriobacteriale*s (I) *Peptostreptococcales-Tissierellales* (J) *S085* (K) *Muribaculaceae* (L) *p__unidentified_Bacteria;f__Eggerthellaceae* (M) *Peptostreptococcaceae*. Data are presented as means ± SD, and analyzed using the one-way ANOVA test with Tukey method. ^#^*P*< 0.05, ^##^*P* < 0.01, ^###^*P* < 0.001 compared with ND group; ^*^*P*< 0.05, ^**^*P* < 0.01, ^***^*P* < 0.001 compared with HFD group.


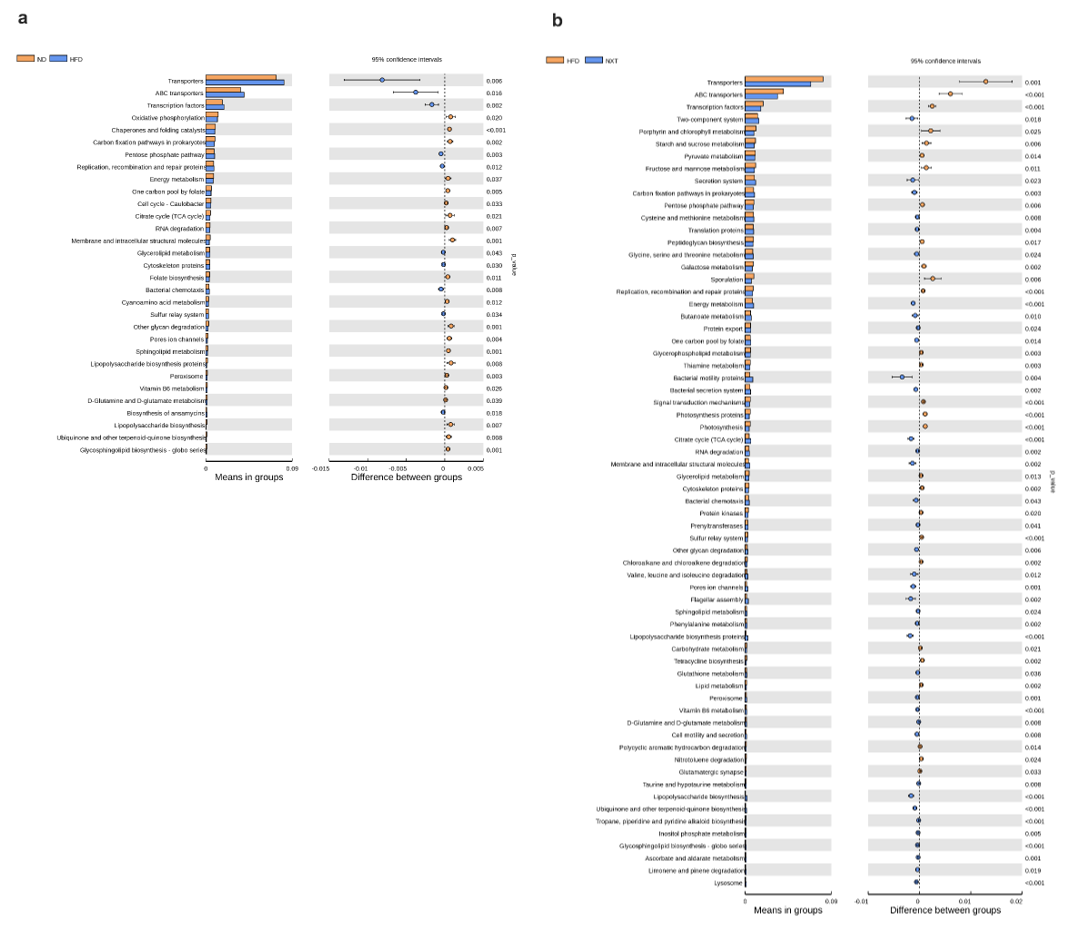


**Supplementary Figure 3.** Metabolic pathways predicted by PICRUST analysis and T-test in three groups. (A) Level 3 predicted data of ND and HFD (B) Level 3 predicted data of HFD and NXT.
